# Genoppi is an open-source software for robust and standardized integration of proteomic and genetic data

**DOI:** 10.1038/s41467-021-22648-5

**Published:** 2021-05-10

**Authors:** Greta Pintacuda, Frederik H. Lassen, Yu-Han H. Hsu, April Kim, Jacqueline M. Martín, Edyta Malolepsza, Justin K. Lim, Nadine Fornelos, Kevin C. Eggan, Kasper Lage

**Affiliations:** 1 Stanley Center at Broad Institute of MIT and Harvard, Cambridge, MA USA; 2 Department of Stem Cell and Regenerative Biology, Harvard University, Cambridge, MA USA; 3 Department of Surgery, Massachusetts General Hospital, Boston, MA USA; 4 Wellcome Centre for Human Genetics, Nuffield Department of Medicine, University of Oxford, Oxford, UK; 5 Department of Computer Science, Johns Hopkins University, Baltimore, MD USA; 6 Massachusetts Institute of Technology, Cambridge, MA USA; 7 Institute of Biological Psychiatry, Mental Health Centre Sct. Hans, Mental Health Services Copenhagen, Roskilde, Denmark

**Keywords:** Protein-protein interaction networks, Proteome informatics, Amyotrophic lateral sclerosis

## Abstract

Combining genetic and cell-type-specific proteomic datasets can generate biological insights and therapeutic hypotheses, but a technical and statistical framework for such analyses is lacking. Here, we present an open-source computational tool called Genoppi (lagelab.org/genoppi) that enables robust, standardized, and intuitive integration of quantitative proteomic results with genetic data. We use Genoppi to analyze 16 cell-type-specific protein interaction datasets of four proteins (BCL2, TDP-43, MDM2, PTEN) involved in cancer and neurological disease. Through systematic quality control of the data and integration with published protein interactions, we show a general pattern of both cell-type-independent and cell-type-specific interactions across three cancer cell types and one human iPSC-derived neuronal cell type. Furthermore, through the integration of proteomic and genetic datasets in Genoppi, our results suggest that the neuron-specific interactions of these proteins are mediating their genetic involvement in neurodegenerative diseases. Importantly, our analyses suggest that human iPSC-derived neurons are a relevant model system for studying the involvement of BCL2 and TDP-43 in amyotrophic lateral sclerosis.

## Abstract (web summary)

Genetic variation can impact protein complexes and interaction networks, but reconciling genetic and proteomic information remains challenging. To address this need, the authors develop Genoppi —a computational tool for integrating genetics and cell-type-specific proteomics data.

## Introduction

Large-scale genetic datasets, such as those obtained from genome-wide association studies (GWAS) or exome sequencing are becoming increasingly available. Simultaneously, advanced proteomic technologies can generate high-quality cell- and tissue-specific quantitative proteomic data (e.g., from immunoprecipitations followed by tandem mass spectrometry [IP-MS/MS]^1,2^ or whole-proteome analyses of cells or tissues^3^). Integration of genomic and proteomic datasets has revealed that genetic variation implicated in rare and common diseases often manifests at the proteome level, for example, by impacting protein complexes or cellular networks^4^. Data from genetics and cell-type-specific quantitative proteomics, therefore, have the potential to inform each other and lead to key molecular and biological insights. However, even for experts in these fields, the relevant data types are often not interoperable. This creates a bottleneck for functionally interpreting genetic data and dissecting the molecular biology of human diseases. In the longer term, difficulties in reconciling these data types will hamper efforts towards gaining mechanistic insights from genetic data and designing therapeutic interventions. To enable the robust, standardized, and intuitive integration of data from genetics and cell-type-specific quantitative proteomics, we have developed an open-source computational tool named Genoppi^5^ (web application: lagelab.org/genoppi, R package source code: github.com/lagelab/Genoppi), which we apply to 16 cell-type-specific protein–protein interaction datasets to illustrate the functionalities of the tool.

## Results

### Genoppi enables integration of proteomic and genetic data

**Fig. 1:**
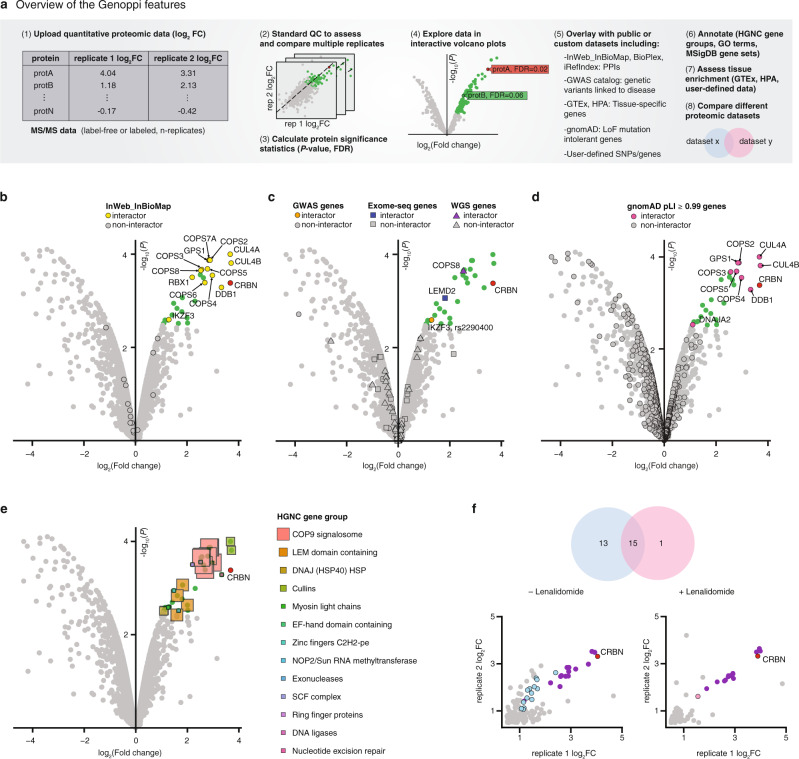
Overview of Genoppi. **a** Overview of the Genoppi features. **b** Volcano plot of published CRBN interaction data in MM1S multiple myeloma cells versus control samples. The *x*-axis shows the log_2_ FC of each identified protein and the *y*-axis the corresponding −log_10_ *P* value. The bait protein (CRBN) is marked in red; statistically significant interactors with log_2_ FC > 0 and FDR ≤ 0.1 are in green; non-interactors that do not pass this threshold are in gray. Known interactors of CRBN in InWeb_InBioMap are marked by black border circles; those significant in the experimental data are highlighted in yellow (overlap enrichment *P* = 1.1e − 21, from one-tailed hypergeometric test). **c** The volcano plot from (**b**) is overlaid with genetic data. Proteins encoded by genes mapped from acute lymphoblastic leukemia GWAS SNPs (GWAS genes), significantly mutated genes identified through exome sequencing in multiple myeloma (Exome-seq genes), or recurrently mutated *cis*-regulatory elements identified via whole-genome sequencing in multiple myeloma (WGS genes) are marked by black border circles, squares, or triangles, respectively; those significant in the experimental data are highlighted in orange, blue, or purple, respectively. Overlap enrichment was not calculated since part of this gene list was mapped from GWAS SNPs using linkage disequilibrium information. **d** The volcano plot from (**b**) is overlaid with proteins intolerant of LoF mutations in gnomAD. Proteins encoded by genes with pLI scores ≥ 0.99 are marked by black border circles; those significant in the experimental data are highlighted in magenta (overlap enrichment *P* = 0.087, from one-tailed hypergeometric test). **e** The volcano plot from (**b**) is overlaid with HGNC gene group annotations (square markers) for the significant interactors. Marker size scales with the number of interactors assigned to each group. **f** Illustration of Genoppi’s ability to make comparisons between proteomic experiments under different genetic or pharmaceutical perturbations; in this case, the comparison of CRBN interactors in untreated (−Lenalidomide) versus lenalidomide-treated (+Lenalidomide) MM1S cells. Top: Venn diagram representing the overlap of significant (log_2_ FC > 0 and FDR ≤ 0.1) interactors between the two conditions. Bottom: scatter plots showing log_2_ FC of identified proteins in two replicates (*x*- and *y*-axis, respectively) for each condition. Interactors shared between conditions are shown in purple; interactors unique to each condition are in blue or pink, respectively. FC fold change, MS mass spectrometry, QC quality control, FDR false discovery rate, PPI protein–protein interaction, LoF loss-of-function, SNP single-nucleotide polymorphism. Source data are provided as a Source Data file.

Given log_2_ fold change (FC) values between studied conditions (e.g., bait versus control IPs) for multiple experimental replicates, Genoppi identifies proteins with statistically significant log_2_ FC by applying user-defined thresholds, displays the data in scatter and volcano plots, and provides options for integrating the results with other forms of data (Fig. 1a, “Methods,” and Supplementary Note 1). Genoppi can quality control (QC) a protein interaction dataset^6^ by testing whether it is enriched for previously known interaction partners compiled from InWeb_InBioMap (InWeb; includes data from > 40,000 scientific articles^7,8^; Fig. 1b), iRefIndex^9^, or BioPlex^10,11^. Genoppi further allows the user to subset the protein interactions in these databases based on the provided confidence metrics (Supplementary Note 1). The ability to automatically integrate these databases with experimental data in real-time makes it easy to distinguish between published versus newly identified interaction partners of a protein of interest, thus eliminating the need to extensively interrogate the literature on a case-by-case basis.

Genoppi can also intersect and co-visualize proteomic data with various types of data derived from population genetic studies. One such data type is a list of single-nucleotide polymorphisms (SNPs) derived from GWAS of a disease of interest. Genoppi automatically uses linkage disequilibrium (LD) information from the 1000 Genomes Project^12^ to identify proteins in a proteomic dataset that are encoded by genes in LD loci of NHGRI-EBI GWAS catalog^13^ or user-defined SNPs^14^ (Fig. 1c and “Methods”). In addition, exome and whole-genome sequencing have been increasingly used to identify genes that have a significant burden of rare mutations in individuals with a particular disease compared to healthy controls. Genoppi is designed to enable the identification of sets of proteins in a proteomic dataset based on such user-defined gene lists^15^ (Fig. 1c). Genoppi can also incorporate gene constraint data from gnomAD^16^ to label proteins intolerant to loss-of-function mutations (Fig. 1d). When these external datasets are integrated with proteomic results, overlaps are displayed in a number of user-defined ways (as Venn diagrams or superimposed on volcano plots), and statistically tested when appropriate (“Methods” and Supplementary Note 1).

Another feature of Genoppi is the annotation of proteomic data with gene sets from several databases, including HGNC^17^, GO^18,19^, and MSigDB^20,21^, to visually identify groups of proteins that may be overrepresented in a particular dataset (Fig. 1e). Genoppi can also perform tissue enrichment analysis using sets of tissue-specific genes derived from RNA or protein expression data in GTEx^22,23^ or the Human Protein Atlas (HPA)^24^ (Supplementary Fig. 1). This feature can be applied to sets of tissue- or cell-type-specific genes uploaded by the user (e.g., cell-type-specific genes identified from single-cell RNA sequencing). Finally, it is possible to perform head-to-head comparisons of proteomic experiments performed under different conditions using Genoppi. For example, interaction experiments that were executed with and without drug treatment^6^, or with the wild-type and mutated versions of a protein, to elucidate the cellular effects of either pharmaceutical or genetic perturbations in a particular cell type (Fig. 1f). Overall, Genoppi provides various ways to explore proteomic datasets, to guide hypothesis generation, or to inform targeted follow-up experiments.

Genoppi is an open-source software that is easily accessible and flexible to meet data-driven custom needs in the research community and is available both as an R package and as a Shiny application with extensive documentation and exemplar datasets. In particular, the Genoppi web application provides a simple interactive interface with customizable options and the ability to work with a wide variety of quantitative proteomic datasets (e.g., IP-MS/MS analyses, or whole-proteome analyses of cells or tissues). For example, it is possible to dynamically explore a dataset by changing various technical thresholds and visualizing the results in real-time; furthermore, a search function enables the quick identification of proteins of interest in various plots (Supplementary Note 1). Users can also locally download the generated data and plots to share with collaborators. For users interested in building custom analytical pipelines, the R package has extensive documentation and can be leveraged for these purposes. For instance, the users may choose to analyze their data with an alternative statistical method before performing downstream analyses using Genoppi. In summary, Genoppi is a highly flexible platform for analyzing cell-type-specific proteomic datasets and facilitating data sharing in cross-disciplinary collaborations that are now common in both academia and industry. Further details about options, workflows, and analyses can be found in Supplementary Note 1.

### Applying Genoppi to analyze cell-type-specific IP-MS/MS data

To exemplify how analyzing cell-type-specific proteomic data using Genoppi can uncover convergent and divergent disease-relevant biology of the same protein in different cell types, we generated IP-MS/MS data for four proteins of interest (BCL2, TDP-43, MDM2, PTEN; baits, hereafter) in four distinct cell lines (“Methods” and Supplementary Data 1). We chose these four proteins because they play important, but not fully elucidated roles in cancer, neurological disease, and psychiatric conditions.

**Fig. 2:**
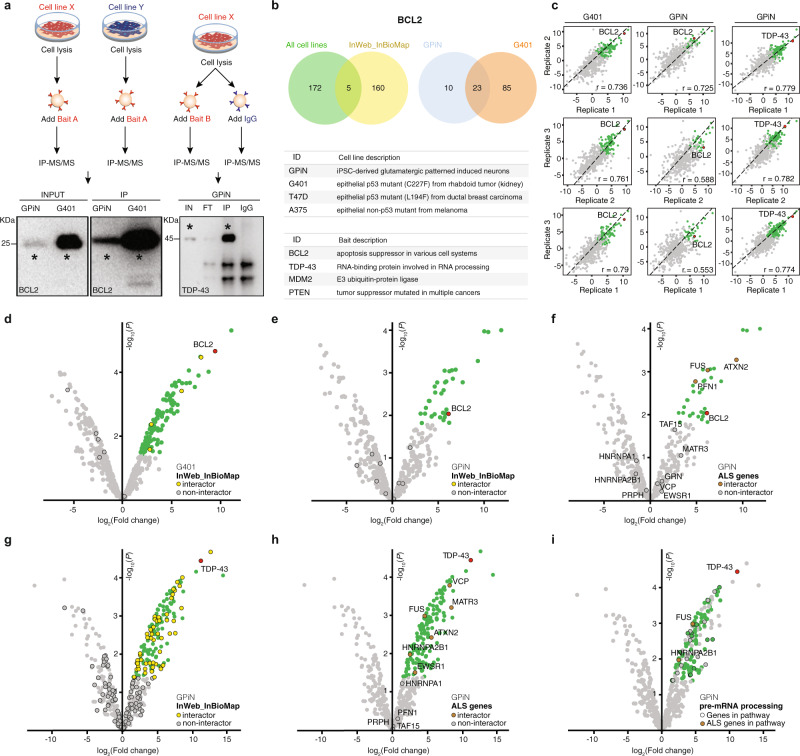
IP-MS/MS analysis through Genoppi. **a** Experimental design and representative western blots of immunoprecipitations prepared for MS/MS analysis. The schematic to the left shows how Bait A (BCL2 as an example) was pulled down in two distinct cell lines (GPiN and G401) and detected in western blots carried out on cell lysate input and IP material from both cell lines. The schematic to the right exemplifies the immunoprecipitation of Bait B (TDP-43 as an example) and the parallel addition of nonspecific IgG control to GPiN lysates; a TDP-43 western blot was then performed on the cell lysate input, IP flow-through, IP, and IgG control. Each blot is representative of three IP replicates. Asterisks (*) indicate the band corresponding to each bait (BCL2 or TDP-43). **b** Top: Venn diagrams representing the overlap between BCL2 interactors identified in all cell lines and known InWeb_InBioMap interactors, and the overlap of interactors identified in neurons (GPiN) and a cancer cell line (G401). Bottom: complete list of cell lines and baits used for the experiments. **c** Scatter plots showing the reproducibility of three IP replicates in terms of log_2_ FC correlation for three sets of experiments: BCL2 versus IgG control in G401 cells or GPiNs, and TDP-43 versus IgG control in GPiNs. Pearson’s correlation (*r*) is reported in each plot. **d** BCL2 versus IgG control IP results in G401 cells. The volcano plot is overlaid with known BCL2 interactors in InWeb_InBioMap (overlap enrichment *P* = 0.15). **e**, **f** BCL2 versus IgG control IP results in GPiNs. The volcano plot is overlaid with known BCL2 interactors in InWeb_InBioMap (**e**; overlap enrichment *P* = 1.0) or proteins encoded by ALS genes (**f**; overlap enrichment *P* = 0.041). **g**, **h** TDP-43 versus IgG control IP results in GPiNs. The volcano plot is overlaid with known TDP-43 interactors in InWeb_InBioMap (**g**; overlap enrichment *P* = 0.085) or proteins encoded by ALS genes (**h**; overlap enrichment *P* = 0.046). In plots (**c**–**h**), the bait (BCL2 or TDP-43), interactors (log_2_ FC > 0 and FDR ≤ 0.1), and non-interactors are shown in red, green, and gray, respectively; overlaid proteins are marked by black border circles, and their overlap enrichment *P* values were calculated using one-tailed hypergeometric tests. **i** TDP-43 versus IgG control IP results in GPiNs shown as volcano plot, with the bait (TDP-43) shown in red, interactors (log_2_ FC > 0 and FDR ≤ 0.1) that are GPiN-specific (i.e., not interactors in G401) in green, and other detected proteins in gray. Black border circles indicate interactors in the MSigDB Reactome “processing of capped intron-containing pre-mRNA” pathway; two GPiN-specific interactors in the pathway, FUS and HNRNPA2B1, have been linked to ALS and are highlighted in brown. IN input, FT flow-through, IP immunoprecipitation, IgG Immunoglobulin G isotype control. Source data are provided as a Source Data file.

We executed bait IPs in a human-induced pluripotent stem cell (iPSC)-derived neuronal cell line (glutamatergic patterned induced neurons [GPiNs]^25^) and three cancer cell lines (G401, T47D, and A375; Fig. 2a, c and Supplementary Fig. 2a), along with control experiments using isotype-matched immunoglobulin gamma (IgG; “Methods”), which are commonly included to control for background signal and nonspecific association with antibodies or beads. All bait and IgG control experiments were conducted in triplicate and quantitated by liquid chromatography followed by label-free liquid chromatography-tandem mass spectrometry (LC-MS/MS; “Methods”). Genoppi was used to: (i) QC, analyze, and visualize all IP-MS/MS results; (ii) identify significant interaction partners of each bait in each cell type; (iii) compare significant interaction partners between cell types; and (iv) integrate these data with published genetic datasets for biological discovery and hypothesis generation (Fig. 2, Supplementary Fig. 2, Supplementary Data 2 and 3, and “Methods”).

### BCL2 interactomes in cancer and neuronal cells

We first explored BCL2, which is a well-studied oncoprotein functioning as an apoptosis suppressor in a variety of cell types;^26^ it was also recently shown to be an important regulator of plasticity and cellular resilience during neuronal development^27^. Intriguingly, data from both human brain tissue and animal models of neuropathological conditions suggest a role for BCL2 in cell death regulation in the mature nervous system^28^ and in amyotrophic lateral sclerosis (ALS)^29,30^. However, little is known about the specific role of BCL2 in different human cell types, and insights into its overlapping and differential interaction partners in neurons compared to cancer cell lines could generate actionable biological hypotheses of therapeutic relevance.

Across the four tested cell lines, BCL2 had a total of 177 nonredundant statistically significant interaction partners. Interaction partners of any bait protein will hereafter be defined as proteins with a log_2_ FC > 0 and false discovery rate (FDR) ≤ 0.1 in the experiment compared to the cell-type-matched IgG control (“Methods”), while non-interactors are proteins that do not fulfill either criteria. Among BCL2 interactors, only five were previously reported in InWeb (Fig. 2b and Supplementary Data 3). However, when comparing the BCL2 interaction partners in GPiNs versus a cancer cell line (G401), we found both a large set of shared interactors and interactors unique to each cell line (Fig. 2b). Specifically, 23 out of 33 (69.7%) interactors found in GPiNs were also interactors in G401 cells; conversely, 23 out of 108 (21.3%) interactors found in G401 cells were also interactors in GPiNs (*P* = 7.8e − 11, using a hypergeometric distribution). This indicates that, despite little overlap with InWeb interactors and the identification of many cell-type-specific interactors (10 in GPiNs and 85 in G401 cells), a significant subset of new BCL2 interaction partners (*n* = 23) were common to two different cellular backgrounds, and thus are likely to be true interaction partners that have not been reported before (Supplementary Data 3).

We next determined the correlation between replicate samples (Fig. 2c) and visualized the differential interactions of BCL2 in G401 cells versus GPiNs (Fig. 2d–f). Superimposing known interaction partners from InWeb with proteins detected in our IPs (Fig. 2d, e) shows that the majority of the interaction partners we identified in both cell types were new (104 of 108 in G401 cells and 33 of 33 in GPiNs; Supplementary Data 3).

To test whether the BCL2 interactome was enriched for known cancer driver genes in G401 cells, and for genes involved in neurological disorders in GPiNs, we integrated genetic and proteomic data in Genoppi. We used datasets of cancer driver genes^31^, genes involved in neurodevelopmental delay, autism spectrum disorders (ASDs)^32,33^, or schizophrenia (SCZ)^34^, as well as a curated list of genes involved in ALS^35,36^ (Supplementary Data 4). The statistical analyses compare the enrichment of disease-related proteins among the interactors of the bait protein versus the non-interactors (“Methods”). Thus, the statistical enrichment is always conditional on the proteome being expressed in the tested cell line.

We found that known cancer driver genes were evenly distributed between proteins significantly interacting with BCL2 and other proteins in the G401 immunoprecipitate (i.e., the non-interactors; Supplementary Fig. 3a). In contrast, the BCL2 interactome in neurons was enriched for ALS-associated proteins compared to the overall GPiN immunoprecipitate (*P* = 0.041, using a hypergeometric distribution; Fig. 2f). We further show that ALS-implicated proteins were not enriched among BCL2 interactors found in any of the three cancer cell lines (Supplementary Fig. 3b–d), confirming that a potential connection to ALS is a feature of the neuron-specific interaction partners of BCL2. Genoppi thus points to targeted follow-up experiments to explore the biological implications of newly identified neuron-specific interactors of BCL2.

### TDP-43 interactomes in cancer and neuronal cells

We further set out to investigate a well-established ALS-associated risk factor, TDP-43. TDP-43 is a ubiquitously expressed RNA-binding protein encoded by the gene *TARDBP*. Rare mutations in *TARDBP* have been identified as a cause of familial ALS and frontotemporal dementia (FTD). TDP-43 aggregation is also a common pathological hallmark of both neurodegenerative disorders^37^. To gain insights into neuronal functions of TDP-43, we analyzed the IP-MS/MS data of TDP-43 in GPiNs (Fig. 2c) and found many of its known InWeb interaction partners (*P* = 0.085, using a hypergeometric distribution; Fig. 2g). We also integrated known ALS-associated risk genes with the TDP-43 interactome in GPiNs and show that the experiments in GPiNs recapitulate known interactions between TDP-43 and VCP, MATR3, FUS, ATXN2, HNRNPA2B1, and EWSR1 (*P* = 0.046, using a hypergeometric distribution; Fig. 2h).

In the literature, it is highly debated whether the pathologies related to TDP-43 aggregation are due to altered transcriptional regulation or cell toxicity. Interestingly, a large portion of TDP-43 interactors in GPiNs (including two out of six ALS-associated interactors, FUS and HNRNPA2B1) are involved in RNA metabolism, suggesting that the role of TDP-43 in human neurons is mostly related to transcriptional and post-transcriptional regulation, as highlighted using Genoppi’s gene set annotation feature (Fig. 2i). Importantly, none of the ALS-associated interactors involved in transcriptional or post-transcriptional regulation were identified among TDP-43 interactors in any of the cancer lines (Supplementary Data 5), suggesting that in a nonneuronal context, TDP-43 may function through a different molecular mechanism involving a discrete set of cell-type-specific interactors.

TDP-43 has previously been studied in human brain homogenates, which are an aggregate of many different cell types^38^, or in HEK cells^39^ that are less relevant to its role in ALS or FTD. Our Genoppi analyses illustrate that GPiNs recapitulate the known biology of this protein and its physical interactions to a number of known ALS-related proteins. To confirm the interactors identified in GPiNs, we made biological replicates of the TDP-43 IP, quality-controlled 23 interactor-specific antibodies (Supplementary Data 1), and tested 23 interactors identified by IP-MS/MS through western blots (Supplementary Fig. 4 and Supplementary Data 6). We observed a validation rate of 21/23 (or 91.3%) among interactors that span a wide range of log_2_ FC values in our IP-MS/MS data and note that this validation rate is concordant with the FDR cutoff of 0.1 used to separate significant interactors from non-interactors in Genoppi. Importantly, we were able to validate both known TDP-43 interactors reported in InWeb (validation rate of 10/11, or 90.9%; including all five of the tested ALS-relevant proteins) and newly identified interactors (validation rate of 11/12, or 91.7%) with the same degree of success. Among the newly identified interactors, we validated eight out of nine (or 88.9%) interactors found in multiple cell lines and three out of three (or 100%) GPiN-specific interactors. Our validation experiments also span 19 interactors with ≥ two values imputed prior to log_2_ FC calculation (see “Methods”); 17 out of 19 (or 89.5%) could be confirmed, strongly supporting the procedure we used to impute missing values in the mass spectrometry data.

We further executed reciprocal IPs of five of the TDP-43 interactors validated by western blots, including three ALS-relevant proteins (MATR3, ATXN2, and FUS) and two non-ALS proteins (RBMX and PARP1; Supplementary Fig. 5a). We tested for the presence of TDP-43 in these IPs and were able to detect it in 80% of the experiments (Supplementary Note 2 and Supplementary Fig. 5b). The reciprocal IPs further validate our IP-MS/MS data and indicate that human iPSC-derived neurons (GPiNs) can be used as a cell model for studying TDP-43 interactions with proteins involved in neurodegenerative diseases. In the future, it will be of interest to test the functional significance of the convergence of ALS risk genes in the TDP-43 pathway and dissect the role of individual interactions between TDP-43- and ALS-related proteins in the context of transcriptional regulation.

### Cell-type-specific and cell-type-independent interactions

We performed analogous IP-MS/MS experiments and Genoppi analyses of two more proteins (MDM2 and PTEN) that are also hypothesized to have divergent functions in cancer and neurodevelopment. Similar to the observations for BCL2 and TDP-43, we observed a large set of new interaction partners that can be replicated across multiple cell types, as well as a set of cell-type-specific interaction partners that can inform targeted hypotheses and follow-up experiments (Supplementary Note 3, Supplementary Fig. 2b–h, and Supplementary Data 3).

Most published protein–protein interactions to date were derived from large-scale screens using systems that lack human cell-type-specific information (e.g., highly proliferative cell lines such as HEK293 cells, or yeast two-hybrid screens). This means that high-quality interaction experiments executed in specific human cell types can lead to the discovery of many novel interactions. Indeed, when we combined the IP-MS/MS results of BCL2, TDP-43, MDM2, and PTEN across four different cell lines, we found that only 16.6% (144/870) of the interaction partners were reported in InWeb, meaning that up to 83.4% (726/870) of these interactions were new, offering potentially exciting insights into the biology of these proteins. Stratified by protein, 97.2%, 65.8%, 72.0%, and 93.0% of the interaction partners were new for BCL2, TDP-43, MDM2, and PTEN, respectively. Across cell lines, 73.4%, 80.6%, 79.7%, and 78.8% of the interaction partners identified for the four proteins were new in GPiN, G401, T47D, and A375 cells, respectively. We note that, while the statistics here were calculated based on known interactions curated in InWeb, most of the interaction partners were also new according to the iRefIndex or BioPlex database (Supplementary Data 3).

In Fig. 2b, we show that a sizable subset (~70%) of the newly identified interaction partners of BCL2 in GPiNs can be replicated in G401 cells, supporting the biological validity of these interactions even if they have not been previously reported in the literature. Here, we extended the same analysis to all four baits in all possible pairs of cell lines (Supplementary Fig. 6 and Supplementary Data 3), showing that although we identified a large set of new interaction partners for each bait, 54.7% (397/726) of them can be reproduced in multiple cell types. In other words, 37.8% (329/870) of the interactors are not curated in InWeb nor recapitulated in multiple cell types. However, when we performed western blots to validate TDP-43 interactors identified in GPiNs, we observed comparable validation rates between known InWeb interactors (10/11, or 90.9%), non-InWeb interactors found in multiple cell lines (8/9, or 88.9%), and non-InWeb interactors found only in GPiNs (3/3, or 100%; Supplementary Note 2 and Supplementary Data 6). Overall, these observations support the robustness of the new interaction partners we report in this study and illustrate the remarkable opportunity for biological discovery through cell-type-specific proteomic experiments.

Finally, we clustered the four cell lines based on the overlap of interaction partners for each bait between pairs of cell lines, and observed a clear clustering of cancer cells (G401, T47D, and A375) versus GPiNs for three out of four bait proteins (BCL2, TDP-43, and PTEN; Supplementary Fig. 6). This indicates that, as expected, for these proteins the cancer cell interactomes are more similar to each other than to the neuronal interactomes. We pooled the interaction partners of all four bait proteins in the three cancer cell lines (cancer cell interactors, hereafter) and in the neurons (GPiN interactors, hereafter), and tested for enrichment of disease genes in the pooled interactors. While the cancer cell interactors were not enriched for cancer driver genes, GPiN interactors were nominally enriched for the ALS genes (*P* = 0.040, using a hypergeometric distribution).

Together, all four tested proteins exhibited a pattern of both unique interaction partners in each cell type and a statistically significant set of shared interaction partners across cell types. New interaction partners of TDP-43 in GPiNs validated in ~90% of the cases, illustrating the reproducibility of the generated IP-MS/MS data. Our results further suggest that the neuron-specific interactions of BCL2 and TDP-43 link them to genes implicated genetically in ALS and its related biology. Overall, our data indicate that proteins have different groups of interaction partners, some that are cell-type-specific and some that are conserved across many cell types (i.e., cell-type-independent). In the examples we show in this paper, the cell-type-specific protein–protein interactions in a model of human neurons link the tested proteins more strongly to neurodevelopmental diseases than the interactions identified in cancer cell lines.

## Discussion

Several programs are available to the community to analyze raw MS/MS data^40,41^, while other tools, such as ProHits-viz^42^, provide visualization capabilities to summarize protein interaction data as well as communicate quantitative differences between a protein of interest and its potential interaction partners. However, none of these tools focuses specifically on creating a systematic and unified workflow for integrating cell-type-specific quantitative proteomic datasets and genetic information. Genoppi is designed so it can be easily incorporated into any functional genomics pipeline by allowing users to integrate datasets, download the results, and modify the code as needed to extend the software and meet different usability requirements.

Beyond providing Genoppi as an accessible tool, we also apply it to analyze a large set of cell-type-specific protein interaction experiments. Together, these datasets provide insights into the interactome landscape of BCL2, TDP-43, MDM2, and PTEN, and open potential avenues to explore their links to cancers and neurological disorders based on the newly found cell-type-specific interactions. We use the generated data to showcase Genoppi as a resource that can be employed to combine original and published datasets in a simple and clear format, allowing systematic analysis, visualization, and exploration of otherwise heterogeneous proteomic and genetic datasets. Genoppi is available as both an R package and as a Shiny application with documentation and test datasets to get the users started (Supplementary Note 1). We believe that as more genetic and proteomic datasets become available, Genoppi will become an increasingly valuable resource for the scientific community.

## Methods

### Genoppi documentation

A user-friendly documentation of analytical and visualization features implemented in the Genoppi application (v1.0.0) is provided in Supplementary Note 1; documentation for the accompanying R package is available on GitHub (https://github.com/lagelab/Genoppi). This section provides additional technical details for analyses performed by Genoppi.

### Moderated *t* test for identifying significant interaction partners

Given protein log_2_ FC values from ≥ 2 replicates, Genoppi performs a one-sample moderated *t* test from the limma^43^ R package to calculate a two-tailed *P* value and Benjamini–Hochberg FDR for each protein. Limma was originally developed to robustly identify differentially expressed genes in microarray experiments and has since been used on a variety of data types, including proteomic results^44,45^. The empirical Bayes moderated *t* test is used in Genoppi, as it is less sensitive to underestimated sample variances and performs best on small sample sizes compared to the classical *t* test^44^. Throughout the paper, we define significant interaction partners of a bait protein as proteins with log_2_ FC > 0 and FDR ≤ 0.1 in the bait versus IgG IP-MS/MS data, but we note that Genoppi allows the user to adjust these thresholds according to their needs.

### SNP-to-gene mapping

To generate the precalculated data Genoppi uses for SNP-to-gene mapping, we filtered the 1000 Genomes Project^12^ (phase 3) dataset to obtain genotype data for unrelated individuals residing in Utah with Northern and Western European ancestry and SNPs with minor allele frequency ≥ 0.05 and missing rate ≤ 0.1. Pairwise LD between SNPs was calculated using a sliding window of 200 kb, which is the default haplotype block estimation distance used in PLINK^46^ (v1.07). Next, for each SNP, the LD genomic locus was defined as the region covered by other SNPs that have *r*^2^ > 0.6 with the SNP, ± 50 kb on either end. These parameters were chosen to comply with established community standards^34,47–49^. Using the precalculated LD locus boundaries, Genoppi can then identify all Ensembl^50^ protein-coding genes whose coordinates overlap with LD loci given a SNP list of interest. If multiple genes are present in the locus defined by a SNP of interest, all genes are mapped to that SNP.

To verify that SNPs are robustly mapped to genes using the mapping method in Genoppi, we mapped 20 random SNPs to genes using both Genoppi and Disease Association Protein-Protein Link Evaluator^51^ (DAPPLE; v0.18 on https://gpbroad.boardinstitute.org), which is a standard tool for SNP-to-gene mapping. DAPPLE uses the following definition for LD locus of a SNP: “the region containing SNPs with *r*^2^ > 0.5… extended to the nearest recombination hotspot.” Nonetheless, in our comparison test, 100% of genes mapped from SNPs using DAPPLE were analogously mapped using the Genoppi algorithm, illustrating the robustness of our approach.

### Hypergeometric test for assessing overlap enrichment between datasets

One-tailed *P* values are calculated using a hypergeometric distribution to assess the enrichment of overlap between experimental proteomic results and other gene lists, known protein interactors from InWeb^7,8^, iRefIndex^9^, or BioPlex^10,11^, genes intolerant of loss-of-function (LoF) mutations derived from gnomAD^16^, and tissue-specific genes derived from GTEx^22,23^ or HPA^24^. To test overlap with a gene list (e.g., known causal genes for a disease), the “population” (*N*) is defined as all genes encoding proteins identified in the experimental data and “success in population” (*k*) is defined as the subset of *N* that pass the user-defined significance threshold (i.e., genes encoding significant proteins). The “sample” (*n*) contains genes from the gene list that are found in *N* and “success in sample” (*x*) is the overlap between *k* and *n*. Similar definitions apply to test overlap with InWeb, iRefIndex, BioPlex, gnomAD, GTEx, or HPA data, except in this case the “population” (*N*) is the intersection of all genes in the experimental data and in the respective database, while the “sample” (*n*) is the subset of *N* consisting of known interactors for a chosen bait in InWeb, iRefIndex, or BioPlex, LoF-intolerant genes defined using a gnomAD pLI score cutoff, or tissue-specific genes in GTEx or HPA (Supplementary Note 1). The hypergeometric test^52^ is performed to calculate the statistical significance of having a given amount of success in a population. This procedure tests the statistical significance of the overlap between the proteomic and external datasets, while taking into consideration that only a subset of all proteins (and their corresponding genes) are identified in the proteomic data, meaning that the statistical test is conditional on the proteome of the cell type being tested.

The hypergeometric test is not performed for genes derived from the SNP-to-gene mapping feature in Genoppi. Correctly testing the overlap between these genes and the proteomic results is a complicated statistical problem that can easily lead to confounded results and inflated *P* values. Confounders include whether the mapped gene is from a single gene or multigenic locus, the gene length, and its tissue-specific expression pattern, to name a few. To accurately perform this analysis requires a workflow that is dataset-specific and is beyond the scope of Genoppi. To not mislead users, and to ensure that other statistical tests in Genoppi can be considered reliable, we do not test the statistical significance of the overlap between proteomic data and genes mapped from SNPs.

### Cell culture

#### Glutamatergic patterned induced neurons

GPiNs were differentiated to day 31 from a clonally selected induced PSC line (iPS hDFn 83/22 iNgn2#9 [iPS3]) by conditional expression of the neuralizing transcription factor NGN2^25^. Plates were coated with Geltrex (LifeTechnologies, A1413301) adhesion matrix (1:100 in Dulbecco’s modified Eagle’s medium/Nutrient Mixture F-12 (DMEM/F:12); Gibco) and seeded at a density of 40,000 cells cm^−2^ in Stemflex media (Gibco, A3349401) containing 1:400 Genetecin (Thermo Scientific, 10131027) as selective antibiotic and rock inhibitor Y27632 (RI; Stemgent, 04-0012). After expansion, day 0 cells were passaged onto plates coated with Geltrex and adhesion matrix (1:100 in DMEM/F:12) in DMEM/F:12 media containing 1% N2 supplement (Gibco), 1% Glutamax, 0.3% glucose, 0.2% normocin (Invitrogen), 1:10,000 doxycycline hyclate (DOX; Sigma-Aldrich), 1:10,000 LDN-193189 (LDN; Stemgent, 04-0074), 1:5000 XAV939 (XAV; Stemgent, 04-00046), 1:1000 SB431542 (SB; Tocris, 1614), and 1:1000 RI. Day 1 cells were differentiated in DMEM/F:12 media supplemented with 1% N2 supplement, 1% Glutamax, 0.3% glucose, 0.2% normocin, 1:10,000 DOX, 1:20,000 LDN, 1:10,000 XAV, and 1:2000 SB. Day 2 media were DMEM/F:12 supplemented with 1% N2 supplement, 1% Glutamax, 0.3% glucose, 0.2% normocin, and 1:10,000 DOX. On day 3, cells were passaged onto plates coated with Geltrex and adhesion matrix (1:100 in DMEM/F:12) in neurobasal media (Gibco) supplemented with 2% B27 (50×, Gibco), 1% Glutamax, 0.3% glucose, 0.2% normocin, 0.5% Minimum Essential Medium-Eagle with non-essential amino acid (MEM NEAA) (Gibco), 1:10,000 DOX, 1:10,000 brain-derived neurotrophic factor (BDNF), 1:10,000 ciliary neurotrophic factor (CTNF), 1:10,000 glial cell-derived neurotrophic factor (GDNF) (R&D Systems; 248-BD/CF, 257-NT/CF, and 212-GD/CF). On day 6, cells were fed by replacing 50% of the media with neurobasal media supplemented with 2% B27, 1% Glutamax, 0.3% glucose, 0.2% normocin, 0.5% MEM NEAA, 1:10,000 DOX, 1:10,000 BDNF, 1:10,000 CTNF, 1:10,000 GDNF, 2.5 µg mL^−1^ laminin, and 1:10,000 floxuridine (Sigma-Aldrich). On days 9 and 13, cells were fed by replacing 50% of the media with neurobasal media supplemented with 2% B27, 1% Glutamax, 0.3% glucose, 0.2% normocin, 0.5% MEM NEAA, 1:10,000 DOX, 1:10,000 BDNF, 1:10,000 CTNF, 1:10,000 GDNF, and 2.5 µg mL^−1^ laminin. From day 13 onwards, cells were fed every 3–4 days by replacing 50% of the media with neurobasal media supplemented with 2% B27, 1% Glutamax, 0.3% glucose, 0.2% normocin, 0.5% MEM NEAA, and 1:10,000 DOX.

#### Cancer cell lines

We used the following cancer cell lines: A375 (ATCC CRL-1619), a human malignant melanoma cell line exhibiting a wild-type p53 genotype; G401 (ATCC CRL-1441), a kidney rhabdoid tumor cell line with a wild-type p53 genotype; and T47D (ATCC HTB-133), a human breast tumor cell line with a mutated p53 genotype. All cell lines were plated at a density of 40,000 cells cm^−2^ (uncoated plates). Cell maintenance media contained 10% fetal bovine serum and PenStrep (1:1000). A375 cells were cultured in DMEM (Thermo Scientific) and split every 2 days (at a 1:12 ratio), G401 were cultured using McCoy’s 5A (Thermo Scientific) media and split every 2 days (at a 1:10 ratio), and T47D cells were cultured in RPMI no phenol red (Thermo Scientific) media and split every 3 days (at a ratio of 1:4).

All cell lines were incubated at 37 °C, 5% CO_2_. To achieve detachment during passaging, all cell lines were exposed to TrypLE (Thermo Scientific).

### Protein extraction and immunoblotting

Total protein extract was obtained by harvesting cells and either processing them immediately or snap-freezing them on dry ice for storage at −80 °C^53^. In both cases, cell pellets were washed with phosphate-buffered saline (PBS) and resuspended in 10× packed cell volume (PCV) IP lysis buffer (Pierce), with freshly added Halt protease and phosphatase inhibitors (Thermo Scientific). After a 20 min incubation at 4 °C, cells were collected by centrifugation (16,200 × *g*, 20 min, 4 °C) and resuspended in 3× PCV lysis buffer. The concentration of the samples was quantified using the Thermo BCA protein assay, and when not used immediately, samples were stored at −80 °C. Samples for immunoblotting were diluted in 6×SMASH buffer (50 mM Tris HCl pH 6.8, 10% glycerol, 2% sodium dodecyl sulfate (SDS), 0.02% bromophenol blue, 1% β-mercaptoethanol), boiled for 10 min at 95 °C, separated on a NuPAGE 4–12% Bis-Tris Protein Gel (Thermo Scientific), and transferred onto a PVDF membrane (Thermo Scientific) by wet transfer (100 V for 2 h). Membranes were blocked by incubation for 1 h at room temperature in 10 mL Tris-buffered saline (TBS) and 0.1% Tween (TBST) with 5% (w/v) Bio-Rad Blotting-grade Blocker. Blots were incubated overnight at 4 °C with the primary antibody (1:1000), washed three times for 10 min with TBST, and incubated for 45 min with the secondary horseradish peroxidase-conjugated antibody. After washing three times for 5 min with TBST, bands were visualized using ECL (GE Healthcare). All antibodies used in this study are listed in Supplementary Data 1.

### Immunoprecipitations

For each individual experiment, 1–2 mg of protein extract was incubated at 4 °C overnight in the presence of 1–2 μg of the relevant antibody. The next day, 50 μL of Protein G beads (Pierce) were added to each sample and incubated at 4 °C for 4 h. Flow-through was collected and beads were washed once with 1 mL lysis buffer (Pierce) supplemented with Halt protease and phosphatase inhibitors (Thermo Scientific), and twice with PBS. Beads were resuspended in 60 μL of PBS and 10% of the volume was employed for immunoblotting, after being boiled in 6×SMASH buffer (50 mM Tris-HCl pH 6.8, 10% glycerol, 2% SDS, 0.02% bromophenol blue, 1% β-mercaptoethanol) for 10 min at 95 °C. The remaining volume was stored at −80 °C and subsequently used for MS analysis.

### Sample preparation for MS

All immunoprecipitated samples (*n* = 48) and IgG controls (*n* = 12) were in PBS buffer on beads. PBS was removed and samples were dissolved in 50 µL TEAB (triethylammonium bicarbonate, 50 mM) buffer, followed by trypsin (Promega) digestion for 3 h at 38 °C. Digested samples were dried to 20 and 10 µL of each sample was injected in the mass spectrometer.

### Mass spectrometry

LC-MS/MS was performed on a Lumos Tribrid Orbitrap Mass Spectrometer (Thermo Scientific) equipped with Ultimate 3000 (Thermo Scientific) nano-high-performance liquid chromatography. Peptides were separated onto a 150-µm inner diameter microcapillary trapping column, packed with ~2 cm of C18 Reprosil resin (5 µm, 100 Å, Dr. Maisch GmbH, Germany), followed by separation on a 50-cm analytical column (PharmaFluidics, Ghent, Belgium). Separation was achieved by applying a gradient from 5 to 27% acetonitrile in 0.1% formic acid for > 90 min at 200 nL min^−1^. Electrospray ionization was enabled by applying a voltage of 2 kV using a home-made electrode junction at the end of the microcapillary column and sprayed from metal tips (PepSep, Denmark). MS survey scan was performed in the Orbitrap, in a range 400–1800 *m*/*z* at a resolution of 6 × 10^4^, followed by the selection of the 20 most intense ions (TOP20) for CID-MS2 fragmentation in the ion trap using a precursor isolation width window of 2 *m*/*z*, automatic gain control setting of 10,000, and a maximum ion accumulation of 100 ms. Singly charged ion species were not subjected to collision-induced dissociation fragmentation. Normalized collision energy was set to 35 V and an activation time of 10 ms. Ions within a 10 p.p.m. *m*/*z* window around ions selected for MS2 were excluded from further selection for fragmentation for 60 s.

Raw data were analyzed with Proteome Discoverer (v2.4; Thermo Scientific). Assignment of MS/MS spectra was performed using the Sequest HT algorithm by searching the data against a protein sequence database including all entries from the Uniprot_Human2018_SPonly database^54^ as well as other known contaminants such as human keratins and common laboratory contaminants. Quantitative analysis between samples was performed by label-free quantitation (LFQ) between different sets of samples. Sequest HT searches were performed using a 10 p.p.m. precursor ion tolerance and requiring each peptide’s N/C termini to adhere with trypsin protease specificity, while allowing up to two missed cleavages. CID-MS2 spectra were searched with 0.5 Da ion tolerance for fragmentation. Methionine oxidation (+15.99492 Da) was set as variable modification. An MS2 spectra assignment FDR of 1% was applied to both proteins and peptides using the Percolator target-decoy database search.

### Proteomic data preprocessing

Starting with protein level LFQ reports, we performed the following preprocessing steps before inputting the data into Genoppi: (1) performed log_2_ transformation and median normalization of protein intensity values in each experimental sample; (2) filtered out contaminants and protein entries supported by < 2 unique peptides; (3) imputed missing protein intensity values in each sample (see details below); (4) calculated log_2_ FC for each pair of replicate samples (e.g., bait versus IgG control). All preprocessed data and subsequent analysis results (average log_2_ FC, *P* value, and FDR calculated in Genoppi) can be found in Supplementary Data 2.

In order to derive log_2_ FC statistics for each protein detected in the proteomic data, we imputed missing protein intensity values in each sample using a well-established approach^55^ prior to calculating log_2_ FC for a pair of samples. Specifically, to replace each missing value in a sample, we randomly sampled from a normal distribution with a mean of *μ* − 1.8*σ* and standard deviation of 0.3*σ*, where *μ* and *σ* are the mean and standard deviation of the observed intensity values in the sample. This procedure works under the assumption that proteins with missing values likely have low intensities below MS detection threshold, and therefore place these proteins in the lower tail of the observed intensity distribution. As positive control examples for this imputation strategy: some of our bait proteins and their known InWeb interaction partners needed to be imputed as they were not detected in the IgG controls; after imputation, they generally showed statistically significant log_2_ FC values that overlaid nicely with the non-imputed log_2_ FC distribution. As further support, we also successfully validated 17 out of 19 (89.5%) TDP-43 interactors with ≥ 2 imputed values prior to log_2_ FC calculation in GPiNs (Supplementary Note 2).

### SAINTexpress analysis

To assess the robustness of the statistical method (moderated *t* test from the limma R package) implemented in Genoppi when applied to our experimental data, we also used an alternative statistical method, SAINTexpress^56^, to identify significant interaction partners of the four bait proteins in the four cell lines (Supplementary Data 7). For each bait in each cell line, we used the intensity data (from protein level LFQ reports) for the bait versus IgG control samples as input to run SAINTexpress, excluding contaminants and protein entries supported by < 2 unique peptides to be consistent with the filtering used for the analogous Genoppi analysis. We assessed the overlap between the significant interactors identified by Genoppi (log_2_ FC > 0 and FDR ≤ 0.1) and by SAINTexpress (BFDR ≤ 0.1; Supplementary Data 7). On average, SAINTexpress identified more significant interactors compared to Genoppi, but ~85% of the interactors identified by Genoppi were also identified by SAINTexpress. This indicates that there is good agreement between the two methods when applied to our experimental data, and that most of the significant interaction partners we identified using Genoppi could be recapitulated using an alternative, established method.

### Lists of disease-associated genes

In order to investigate the overlap between interactors identified in our proteomic data and disease-associated genes, we compiled several gene lists from published genetic studies (Supplementary Data 4). For cancer, we used a list of 260 genes identified by exome sequencing^31^. For neuropsychiatric disease, we aggregated a total of 571 unique genes that have been implicated in ASD or SCZ. This list includes ASD genes identified by exome sequencing^32^ and mapped from genome-wide significant GWAS index SNPs^33^ using Genoppi’s SNP-to-gene mapping framework. For SCZ, we mapped genome-wide significant GWAS regions^34^ to genes overlapping these regions. For ALS, we curated a list of 53 genes based on literature review^35,36^.

### Reporting summary

Further information on research design is available in the Nature Research Reporting Summary linked to this article.

## Supplementary information

Supplementary Information

Description of Additional Supplementary Files

Supplementary Data 1

Supplementary Data 2

Supplementary Data 3

Supplementary Data 4

Supplementary Data 5

Supplementary Data 6

Supplementary Data 7

Reporting Summary

## Data Availability

Raw IP-MS/MS data generated in this study have been deposited to the ProteomeXchange Consortium via the PRIDE^57^ partner repository with the dataset identifier PXD022667; processed IP-MS/MS data are available in Supplementary Data 2. External databases/datasets used in this study include: UniProt [https://www.uniprot.org], InWeb_InBioMap [https://inbio-discover.intomics.com/#downloads], iRefIndex [https://irefindex.vib.be/wiki/index.php/iRefIndex], BioPlex [https://bioplex.hms.harvard.edu], 1000 Genomes Project [phase 3; https://www.internationalgenome.org], Ensembl [GRCh37; https://www.ensembl.org/Homo_sapiens/Info/Index], NHGRI-EBI GWAS catalog [https://www.ebi.ac.uk/gwas/], gnomAD [https://gnomad.broadinstitute.org], Human Protein Atlas [https://www.proteinatlas.org], GTEx [https://gtexportal.org/home/] tissue-specific genes from Finucane et al.^22^ or Jiang et al.^23^, HGNC [https://www.genenames.org], GO [http://geneontology.org], MSigDB [http://www.gsea-msigdb.org/gsea/msigdb/index.jsp], cancer genes from Lawrence et al.^31^, ASD genes from Satterstrom et al.^32^, ASD GWAS loci from Grove et al.^33^, SCZ GWAS loci from PGC^34^, ALS genes from Farhan et al.^35^ and Volk et al.^36^. Source data are provided with this paper.

## Source data

Source Data

## Code availability

Source code and documentation for the Genoppi^5^ (v1.0.0) R package and Shiny application are available on GitHub [https://github.com/lagelab/Genoppi]. Custom Python (v3.8.0) and R (v3.6.3) scripts used to pre-process the IP-MS/MS data and generate plots shown in the figures, respectively, are available from the corresponding authors upon request.

## References

[1] Lundby A (2014). Annotation of loci from genome-wide association studies using tissue-specific quantitative interaction proteomics. Nat. Methods.

[2] Lage K (2014). Protein-protein interactions and genetic diseases: The interactome. Biochim. Biophys. Acta.

[3] Ahmad Y, Lamond AI (2014). A perspective on proteomics in cell biology. Trends Cell Biol..

[4] Viswanathan SR (2018). Genome-scale analysis identifies paralog lethality as a vulnerability of chromosome 1p loss in cancer. Nat. Genet..

[5] Pintacuda, G. et al. Genoppi is an open-source software for robust and standardized integration of proteomic and genetic data. *lagelab/Genoppi: Genoppi v1.0.0*. 10.5281/zenodo.4532375 (2021). 10.1038/s41467-021-22648-5 PMC8110583 33972534

[6] Kronke J (2014). Lenalidomide causes selective degradation of IKZF1 and IKZF3 in multiple myeloma cells. Science.

[7] Lage K (2007). A human phenome-interactome network of protein complexes implicated in genetic disorders. Nat. Biotechnol..

[8] Li T (2017). A scored human protein-protein interaction network to catalyze genomic interpretation. Nat. Methods.

[9] Razick S, Magklaras G, Donaldson IM (2008). iRefIndex: a consolidated protein interaction database with provenance. BMC Bioinform..

[10] Huttlin EL (2015). The BioPlex network: a systematic exploration of the human interactome. Cell.

[11] Huttlin, E. L. et al. Dual proteome-scale networks reveal cell-specific remodeling of the human interactome. Preprint at *bioRxiv*10.1101/2020.01.19.905109 (2020). 10.1016/j.cell.2021.04.011 PMC8165030 33961781

[12] The 1000 Genomes Project Consortium. (2015). A global reference for human genetic variation. Nature.

[13] Buniello A (2019). The NHGRI-EBI GWAS Catalog of published genome-wide association studies, targeted arrays and summary statistics 2019. Nucleic Acids Res..

[14] Wiemels JL (2018). GWAS in childhood acute lymphoblastic leukemia reveals novel genetic associations at chromosomes 17q12 and 8q24.21. Nat. Commun..

[15] Hoang PH (2018). Whole-genome sequencing of multiple myeloma reveals oncogenic pathways are targeted somatically through multiple mechanisms. Leukemia.

[16] Karczewski KJ (2020). The mutational constraint spectrum quantified from variation in 141,456 humans. Nature.

[17] Yates B (2017). Genenames.org: the HGNC and VGNC resources in 2017. Nucleic Acids Res..

[18] Ashburner M (2000). Gene ontology: tool for the unification of biology. The Gene Ontology Consortium. Nat. Genet..

[19] The Gene Ontology Consortium. (2019). The Gene Ontology Resource: 20 years and still GOing strong. Nucleic Acids Res..

[20] Subramanian A (2005). Gene set enrichment analysis: a knowledge-based approach for interpreting genome-wide expression profiles. Proc. Natl Acad. Sci. USA.

[21] Liberzon A (2015). The Molecular Signatures Database (MSigDB) hallmark gene set collection. Cell Syst..

[22] Finucane HK (2018). Heritability enrichment of specifically expressed genes identifies disease-relevant tissues and cell types. Nat. Genet..

[23] Jiang L (2020). A Quantitative Proteome Map of the human body. Cell.

[24] Uhlen M (2015). Proteomics. Tissue-based map of the human proteome. Science.

[25] Nehme R (2018). Combining NGN2 programming with developmental patterning generates human excitatory neurons with NMDAR-mediated synaptic transmission. Cell Rep..

[26] Yip KW, Reed JC (2008). Bcl-2 family proteins and cancer. Oncogene.

[27] Opferman JT, Kothari A (2018). Anti-apoptotic BCL-2 family members in development. Cell Death Differ..

[28] Akhtar RS, Ness JM, Roth KA (2004). Bcl-2 family regulation of neuronal development and neurodegeneration. Biochim. Biophys. Acta.

[29] Pasinelli P (2004). Amyotrophic lateral sclerosis-associated SOD1 mutant proteins bind and aggregate with Bcl-2 in spinal cord mitochondria. Neuron.

[30] Mu X, He J, Anderson DW, Trojanowski JQ, Springer JE (1996). Altered expression of bcl-2 and bax mRNA in amyotrophic lateral sclerosis spinal cord motor neurons. Ann. Neurol..

[31] Lawrence MS (2013). Mutational heterogeneity in cancer and the search for new cancer-associated genes. Nature.

[32] Satterstrom FK (2020). Large-scale exome sequencing study implicates both developmental and functional changes in the neurobiology of autism. Cell.

[33] Grove J (2019). Identification of common genetic risk variants for autism spectrum disorder. Nat. Genet..

[34] Schizophrenia Working Group of the Psychiatric Genomics Consortium. (2014). Biological insights from 108 schizophrenia-associated genetic loci. Nature.

[35] Farhan SMK (2019). Exome sequencing in amyotrophic lateral sclerosis implicates a novel gene, DNAJC7, encoding a heat-shock protein. Nat. Neurosci..

[36] Volk AE, Weishaupt JH, Andersen PM, Ludolph AC, Kubisch C (2018). Current knowledge and recent insights into the genetic basis of amyotrophic lateral sclerosis. Med. Genet..

[37] Prasad A, Bharathi V, Sivalingam V, Girdhar A, Patel BK (2019). Molecular mechanisms of TDP-43 misfolding and pathology in amyotrophic lateral sclerosis. Front. Mol. Neurosci..

[38] Umoh ME (2018). A proteomic network approach across the ALS-FTD disease spectrum resolves clinical phenotypes and genetic vulnerability in human brain. EMBO Mol. Med..

[39] Freibaum BD, Chitta RK, High AA, Taylor JP (2010). Global analysis of TDP-43 interacting proteins reveals strong association with RNA splicing and translation machinery. J. Proteome Res..

[40] Cox J, Mann M (2008). MaxQuant enables high peptide identification rates, individualized p.p.b.-range mass accuracies and proteome-wide protein quantification. Nat. Biotechnol..

[41] Tran NH (2019). Deep learning enables de novo peptide sequencing from data-independent-acquisition mass spectrometry. Nat. Methods.

[42] Knight JDR (2017). ProHits-viz: a suite of web tools for visualizing interaction proteomics data. Nat. Methods.

[43] Ritchie ME (2015). limma powers differential expression analyses for RNA-sequencing and microarray studies. Nucleic Acids Res..

[44] Smyth, G. K. Linear models and empirical bayes methods for assessing differential expression in microarray experiments. *Stat. Appl. Genet. Mol. Biol*. **3**, Article3 (2004). 10.2202/1544-6115.1027 16646809

[45] Agnetti, G., Lindsey M. L. & Foster, D. B. *Manual of Cardiovascular Proteomics* (Springer, 2016).

[46] Purcell S (2007). PLINK: a tool set for whole-genome association and population-based linkage analyses. Am. J. Hum. Genet..

[47] Liu JZ (2010). A versatile gene-based test for genome-wide association studies. Am. J. Hum. Genet..

[48] Nyholt DR (2012). Genome-wide association meta-analysis identifies new endometriosis risk loci. Nat. Genet..

[49] Tang W (2012). Genetic associations for activated partial thromboplastin time and prothrombin time, their gene expression profiles, and risk of coronary artery disease. Am. J. Hum. Genet..

[50] Yates AD (2020). Ensembl 2020. Nucleic Acids Res..

[51] Rossin EJ (2011). Proteins encoded in genomic regions associated with immune-mediated disease physically interact and suggest underlying biology. PLoS Genet..

[52] Feller, W. *An Introduction to Probability Theory and tts Applications* (Wiley, 1968).

[53] Dignam JD, Lebovitz RM, Roeder RG (1983). Accurate transcription initiation by RNA polymerase II in a soluble extract from isolated mammalian nuclei. Nucleic Acids Res..

[54] The UniProt Consortium. (2018). UniProt: the universal protein knowledgebase. Nucleic Acids Res..

[55] Tyanova S (2016). The Perseus computational platform for comprehensive analysis of (prote)omics data. Nat. Methods.

[56] Teo G (2014). SAINTexpress: improvements and additional features in significance analysis of INTeractome software. J. Proteom..

[57] Perez-Riverol Y (2019). The PRIDE database and related tools and resources in 2019: improving support for quantification data. Nucleic Acids Res..

