# Supplementary material for: Genoppi is an open-source software for robust and standardized integration of proteomic and genetic data
Source: Nat Commun. 2021 May 10;12:2580. doi: 10.1038/s41467-021-22648-5 (PMC8110583; doi:10.1038/s41467-021-22648-5)
Supplement: Supplementary file 1 — Supplementary Information [file 41467_2021_22648_MOESM1_ESM.pdf]

Supplementary Information

Genoppi is an open-source software for robust and standardized integration of proteomic and genetic data

Greta Pintacuda*, Frederik H. Lassen*, Yu-Han H. Hsu*, April Kim*, Jacqueline M. Martín, Edyta Malolepsza, Justin K. Lim, Nadine Fornelos, Kevin C. Eggan, Kasper Lage

*These authors contributed equally.

Table of contents

Supplementary Note 1 pg. 2
- Getting started pg. 2
- Input format pg. 3
- Basic plotting pg. 5
- Integrated plotting pg. 7
- Gene set annotations pg. 13
- Tissue enrichment pg. 15
- Multiple files comparison pg. 18
- Downloads pg. 21

Supplementary Note 2 pg. 22
Supplementary Note 3 pg. 24
Supplementary Figure 1 pg. 26
Supplementary Figure 2 pg. 27
Supplementary Figure 3 pg. 29
Supplementary Figure 4 pg. 30
Supplementary Figure 5 pg. 31
Supplementary Figure 6 pg. 32
Supplementary References pg. 33

Supplementary Note 1. Protocol for the Genoppi (v1.0.0) web application.

Getting started

Genoppi is an open-source software for performing quality control and analyzing quantitative human proteomic data. Genoppi streamlines the integration of user-inputted proteomic data with external datasets such as known protein-protein interactions in published literature, data from population genetic studies, gene set annotations, tissue-specific RNA or protein expression, or other user-defined inputs. This protocol provides documentation for using the interactive Genoppi web application, which is available at www.lagelab.org/genoppi. Descriptions for external datasets incorporated into Genoppi are provided in the "Data Documentation" page of the application. Source code for the application and the stand-alone Genoppi R package can be downloaded at github.com/lagelab/Genoppi for local installation.

Please direct questions and comments to Kasper Lage (lage.kasper@mgh.harvard.edu).

Input format

Genoppi can be used to analyze quantitative proteomic data that contain protein $\log_2$ fold change (FC) values between studied conditions, such as bait vs. control immunoprecipitations followed by mass spectrometry (IP-MS). Protein quantification results generated using labeling-based (e.g., iTRAQ, TMT, or SILAC) or label-free MS methods can be inputted into Genoppi following the input file format described below.

The input file must be a tab-delimited text file. At minimum, the file must contain three columns, with one column specifying protein identifiers and two columns listing protein $\log_2$ FC values for two or more experimental replicates. More specifically:

Column 1: protein identifiers as either HUGO Gene Nomenclature Committee[1] (HGNC) [www.genenames.org] approved symbols (with “gene” as column header), or UniProt[2] [www.uniprot.org] accession numbers (with “accession_number” as column header).

Columns 2, 3, (+ optional additional columns): $\log_2$ FC values for ≥ two replicates, with “rep1”, “rep2”, and so on as column headers for the replicates.
OR
Columns 2, 3, 4: average $\log_2$ FC across replicates (“logFC”) with corresponding *P*-value (“pvalue”) and false discovery rate (“FDR”) calculated using a statistical test (e.g., a moderated t-test).

Missing values are not allowed; any rows with missing values would be disregarded with no error message.

Examples of accepted input format with correct column headers:

1. HGNC symbol and $\log_2$ FC for two replicates

gene	rep1	rep2
FOXP2	-0.496	-0.546
RB1	0.402	0.265
SHH	0.08	0.104

2. UniProt accession number and log_2 FC for three replicates

accession_number	rep1	rep2	rep3
O15409	-0.496	-0.546	-0.447
P06400	0.402	0.265	0.410
Q15465	0.08	0.104	0.125

3. HGNC symbol and pre-calculated results of statistical test

gene	logFC	pvalue	FDR
FOXP2	-0.521	1.64e-3	4.14e-3
RB1	0.334	0.0118	0.0189
SHH	0.092	0.211	0.242

For Mac users exporting data from Excel format, please convert it to text file by selecting “File” > “Save As…” > “File Format” > “Tab delimited Text (.txt)”. This would avoid generating a file that terminates each line with a carriage return character, which is incompatible with subsequent analysis in Genoppi.

Basic plotting

Screenshot 1 illustrates the basic user interface of the Genoppi application. After the user uploads a "Single File" input in the left panel (**Screenshot 1a**), the "Basic plotting" module will generate an interactive volcano plot, depicting the average $\log_2$ FC of proteins on the x-axis and the $-\log_{10}$ *P*-value on the y-axis. If $\log_2$ FC values from ≥ two replicates are provided in the input file, a moderated *t*-test from the limma[3] R package is applied to calculate the average $\log_2$ FC, nominal *P*-value, and FDR; otherwise, Genoppi uses the user-supplied statistics to generate the plot. In addition, a scatter plot showing replicate $\log_2$ FC correlation is generated if the input file includes separate replicates; when there are > 2 replicates, the user can select from a drop-down menu to show the scatter plot corresponding to each pair of replicates (**Screenshot 1b**).

In the default coloring scheme, significant proteins with $\log_2$ FC ≥ 0 and FDR ≤ 0.1 are in green, and other detected proteins are in grey. The user can change the colors (**Screenshot 1c**) or modify the significance threshold for defining significant proteins based on different FDR, *P*-value, and $\log_2$ FC cutoffs (**Screenshot 1d**). The adjustable cutoffs allow Genoppi to account for various types of proteomic experiments. For instance, when identifying the interactome of a bait protein compared to control, the user should look for significant proteins with positive $\log_2$ FC; when identifying proteins with differential abundance in two experimental conditions, significant proteins with either positive or negative $\log_2$ FC are both of interest. The "Summary" box shows the number of significant proteins (and total number of detected proteins) based on the specified threshold, as well as the correlation between replicates when appropriate. Hovering over each protein's data point in either the volcano or scatter plot would show its corresponding HGNC symbol. The user can also query specific HGNC symbols to label bait and other proteins in the plots (**Screenshot 1e**).

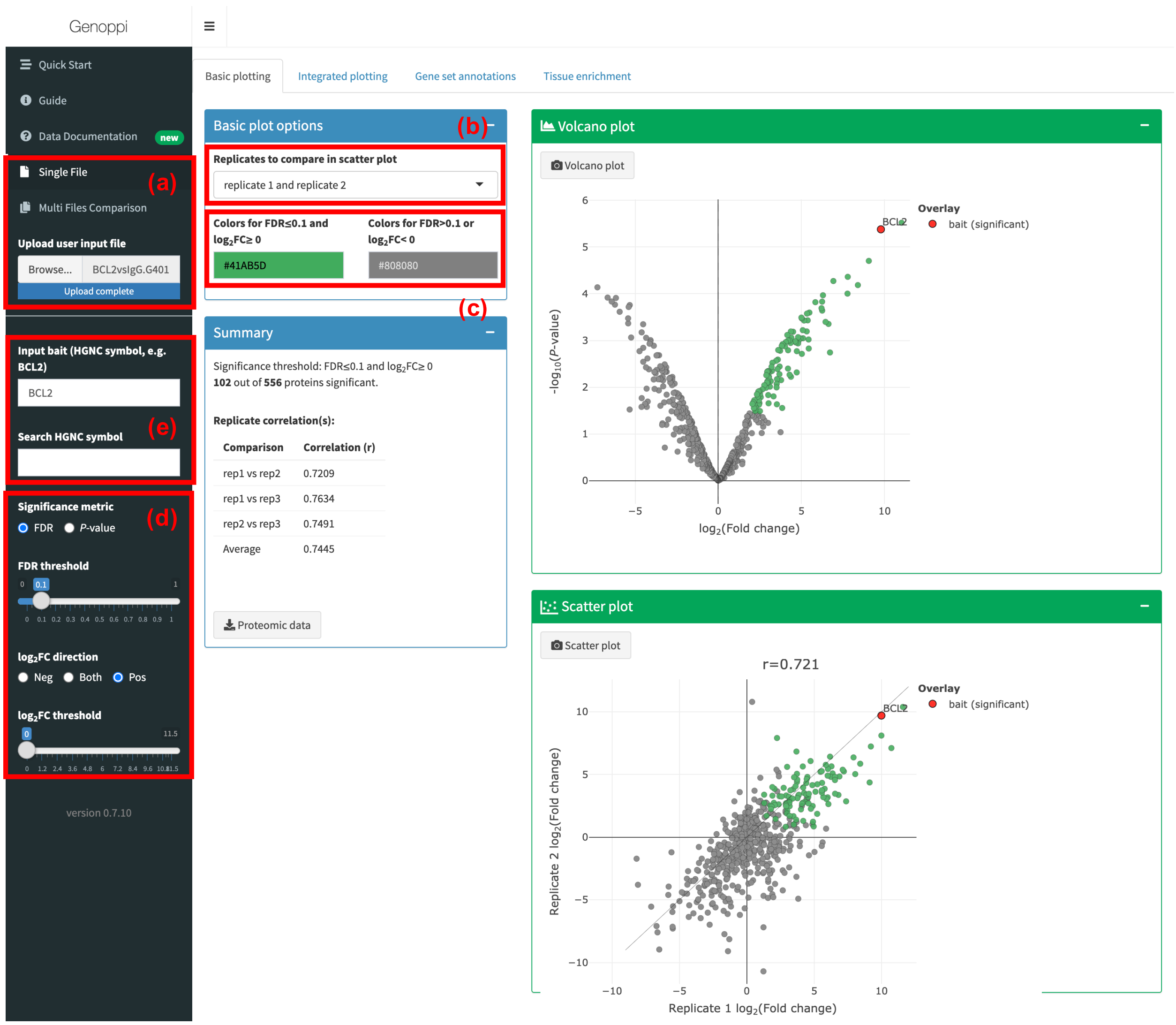

Screenshot 1. Basic plotting interface showing volcano and replicate correlation scatter plots generated from input proteomic data.

Integrated plotting

In the "Integrated plotting" module, Genoppi enables integration of the proteomic data with data from the InWeb_InBioMap[4, 5], iRefIndex[6], BioPlex[7, 8], NHGRI-EBI GWAS catalog[9], gnomAD[10], GTEx[11, 12], Human Protein Atlas[13] (HPA), or user-uploaded SNP or gene lists.

InWeb_InBioMap, iRefIndex, or BioPlex

Genoppi can overlay the proteomic data with published human protein-protein interactions in PPI databases, including InWeb_InBioMap (InWeb) [www.intomics.com/inbio/map.html#downloads], iRefIndex [irefindex.vib.be/wiki/index.php/iRefIndex], and BioPlex [bioplex.hms.harvard.edu]. This integration enables the user to easily distinguish new interactions from those already reported in the literature. The user can search for known interactors of a specific protein in the selected database to visualize their overlap with significant proteins in the proteomic data in an overlaid volcano plot (**Screenshot 2a**). In addition, the user can choose to subset the published interactions based on the confidence metric provided by each database. For instance, InWeb interactors can be subsetted to "gold-standard" interactors curated from pathway databases, or "high-confidence" interactors with high confidence scores in the database, thereby excluding noisier data in the literature. In the volcano plot, published interactors (as well as other data types described below) detected in the proteomic data are labeled using an adjustable color and shape scheme (**Screenshot 2b**).

GWAS catalog

Genoppi can perform SNP-to-gene mapping for SNPs found in the 1000 Genomes Project[14] [www.internationalgenome.org], using pre-calculated pairwise linkage disequilibrium (LD) measures between SNPs to identify all genes in LD regions. Therefore, the user can query diseases and traits found in the NHGRI-EBI GWAS catalog [www.ebi.ac.uk/gwas] to identify genes mapped from published trait-associated SNPs in the catalog (**Screenshot 2c**). Proteins encoded by the mapped genes would be labeled in the interactive volcano plot, and hovering over each of these proteins would show the SNP(s) that map to it.

gnomAD

Genoppi can identify proteins encoded by genes that are likely intolerant of loss-of-function (LoF) mutations using constraint data from gnomAD [gnomad.broadinstitute.org]. The user can label

proteins with pLI scores (i.e. probability of intolerance to LoF mutations) greater than an adjustable threshold to visualize the most intolerant proteins in the overlaid volcano plot (**Screenshot 2d**).

GTEx or HPA

Genoppi can identify proteins encoded by tissue-specific genes derived from one of three GTEx [gtexportal.org] or HPA [www.proteinatlas.org] datasets: (1) "GTEx - RNA": tissue-specific genes defined by Finucane *et al.*[11] using GTEx RNA-seq data, (2) "GTEx - protein": tissue-enriched genes defined by Jiang *et al.*[12] using GTEx protein expression data, and (3) "HPA - RNA": tissue-elevated genes defined by Uhlén *et al.*[13] using HPA RNA-seq data. After picking one of the datasets in a drop-down menu, the user can select tissue(s) found in the dataset to visualize the corresponding tissue-specific proteins in the overlaid volcano plot (**Screenshot 2e**).

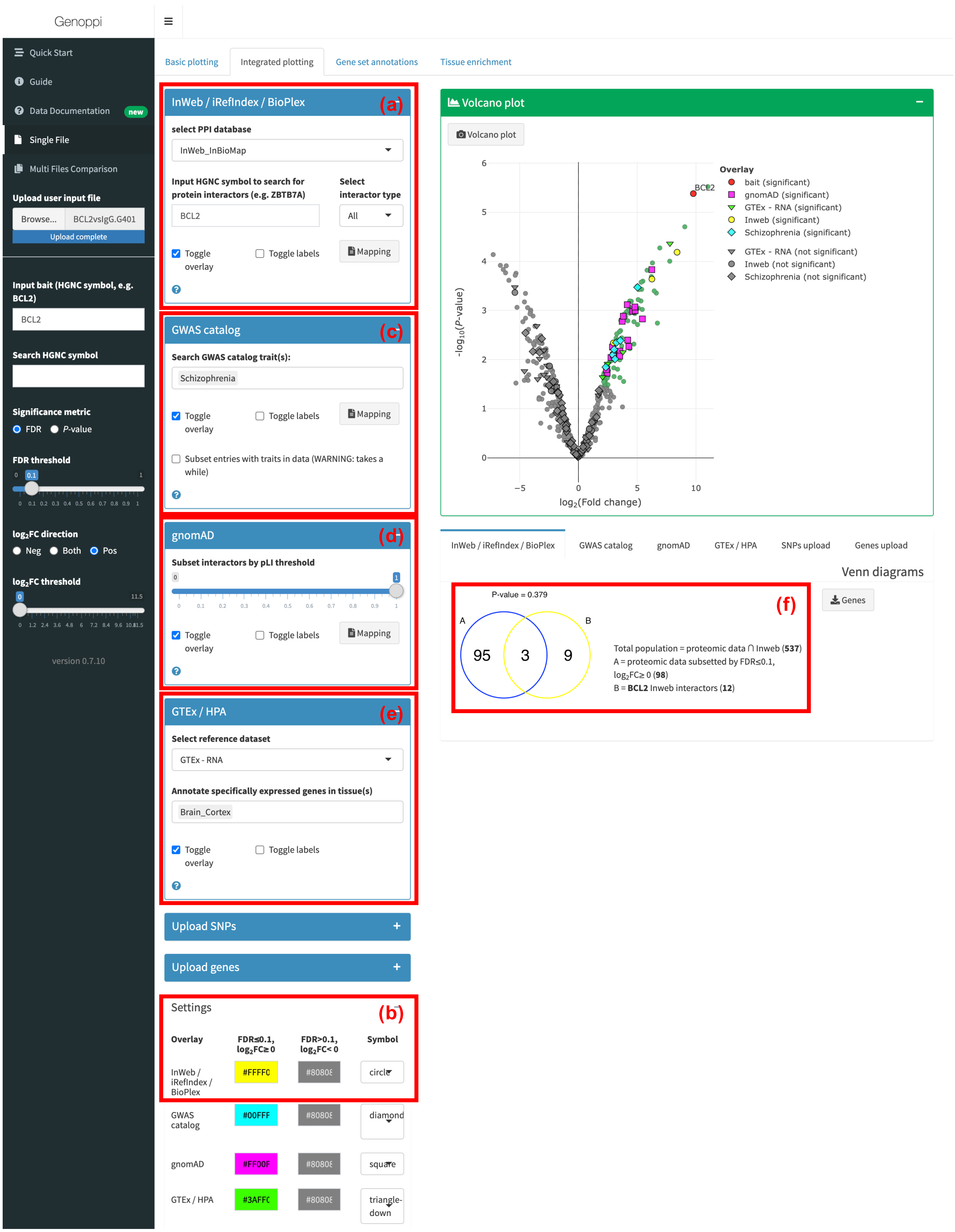

Screenshot 2. Integrated plotting interface showing integration of proteomic data with InWeb, GWAS catalog, gnomAD, and GTEx or HPA data.

Upload SNPs or genes

Besides incorporating the public datasets described above, the user may also upload one or more custom SNP or gene lists (e.g. disease-causing genes curated from literature review or genes implicated by gene-based burden testing) to assess their overlaps with the proteomic data (**Screenshot 3a**). As described in the "GWAS catalog" section, uploaded SNPs would be mapped to genes in LD using Genoppi's built-in SNP-to-gene mapping functionality. The SNP list(s) must be uploaded as a tab-delimited plain text file containing two columns: "listName" (name for each list) and "SNP" (rsID). For example:

```
listName    SNP
List1       rs848132
List1       rs244285
List1       rs10757278
List2       rs3892097
List2       rs539515
```

Similarly, the gene list(s) must be uploaded as a tab-delimited text file consisting of two columns: "listName" (name for each list) and "gene" (HGNC symbol). For example:

```
listName    gene
ListA       SHH
ListA       UBC
ListB       FOXP2
ListB       RB1
ListB       KRAS
```

In the "Integrated plotting" module, the user may input any combination of InWeb interaction partners, GWAS catalog mapped genes, gnomAD constrained genes, GTEx or HPA tissue-specific genes, and custom SNP and gene lists. The resulting volcano plot would highlight all identified proteins from these inputs. Overlaying multiple datasets could result in a densely labeled plot, in which case the user can choose to remove the overlay or the protein text labels for each data type using the "Toggle overlay" or "Toggle labels" option, respectively (**Screenshot 3b**).

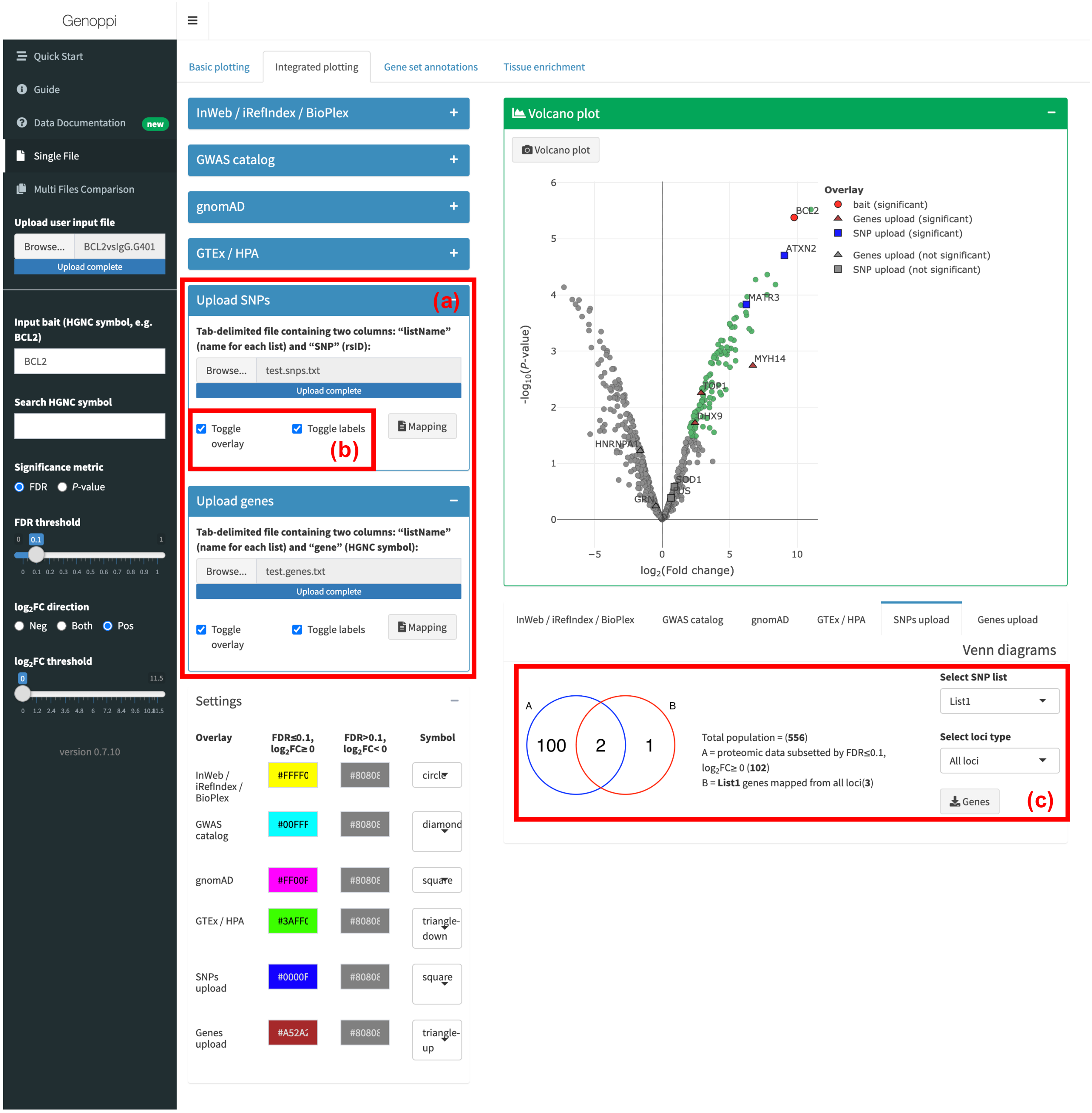

Screenshot 3. Integrated plotting interface showing integration of proteomic data with user-uploaded SNP and gene lists.

Venn diagrams

In the “Integrated plotting” module, Genoppi also summarizes the overlaps between the significant proteins in the proteomic data and the various data types described above using Venn diagrams. When showing overlap with PPI database interactors, gnomAD constrained genes, GTEx or HPA tissue-specific genes, or user-uploaded gene lists, Genoppi also assesses the overlap enrichment by calculating a hypergeometric *P*-value, which is displayed above the Venn diagram (**Screenshot 2f**). For genes mapped from GWAS catalog or user-uploaded SNP lists, this calculation is not performed as the statistic is not robust when each SNP could be mapped to multiple genes in LD. In addition, for user-uploaded SNP lists (which should contain only independent SNPs), Genoppi generates three Venn diagrams to show the overlap of significant proteins with all mapped genes, genes in single-gene loci, or genes in multi-gene loci, respectively; these diagrams can be selected using a drop-down menu (**Screenshot 3c**).

If the user uploads multiple SNP or gene lists, Venn diagrams and overlap statistics for each list would be generated separately for each list, and the individual list results can be accessed through clicking on the list name in a drop-down menu (**Screenshot 3c**). Note that when a bait protein has been indicated in the “Input bait” search box in the left panel, Genoppi would exclude the bait when calculating the numbers and statistics in the *Venn diagrams* section.

Gene set annotations

In the "Gene set annotations" module, Genoppi enables annotation of the proteomic data with gene sets from various databases, including HGNC gene groups, Gene Ontology[15, 16] (GO) [geneontology.org] terms (molecular function, cellular component, and biological process), and MSigDB[17, 18] [www.gsea-msigdb.org/gsea/msigdb/index.jsp] gene sets (H and C1-C7 collections), allowing the user to explore the diversity of protein functions in the proteomic results.

The user can annotate significant proteins in their volcano plot by selecting a collection of gene sets from a drop-down menu (**Screenshot 4a**). Proteins belonging to different gene sets are annotated using square markers of distinct colors; the marker size is scaled with the frequency of each gene set (i.e. number of proteins assigned to each set), providing quick visualization of overrepresentation trends in the data. The volcano plot can display up to 100 most recurrent gene sets at once; the user can further filter these top gene sets using the frequency slider (**Screenshot 4a**). Hovering over each marker in the resulting volcano plot would show the protein's gene set annotations. Alternatively, the table below the volcano plot lists all the gene set annotations without the 100 gene sets limitation. Finally, the user can also query specific gene sets using a search box (**Screenshot 4b**), and the proteins belonging to the queried sets would be labeled with diamond markers in the volcano plot.

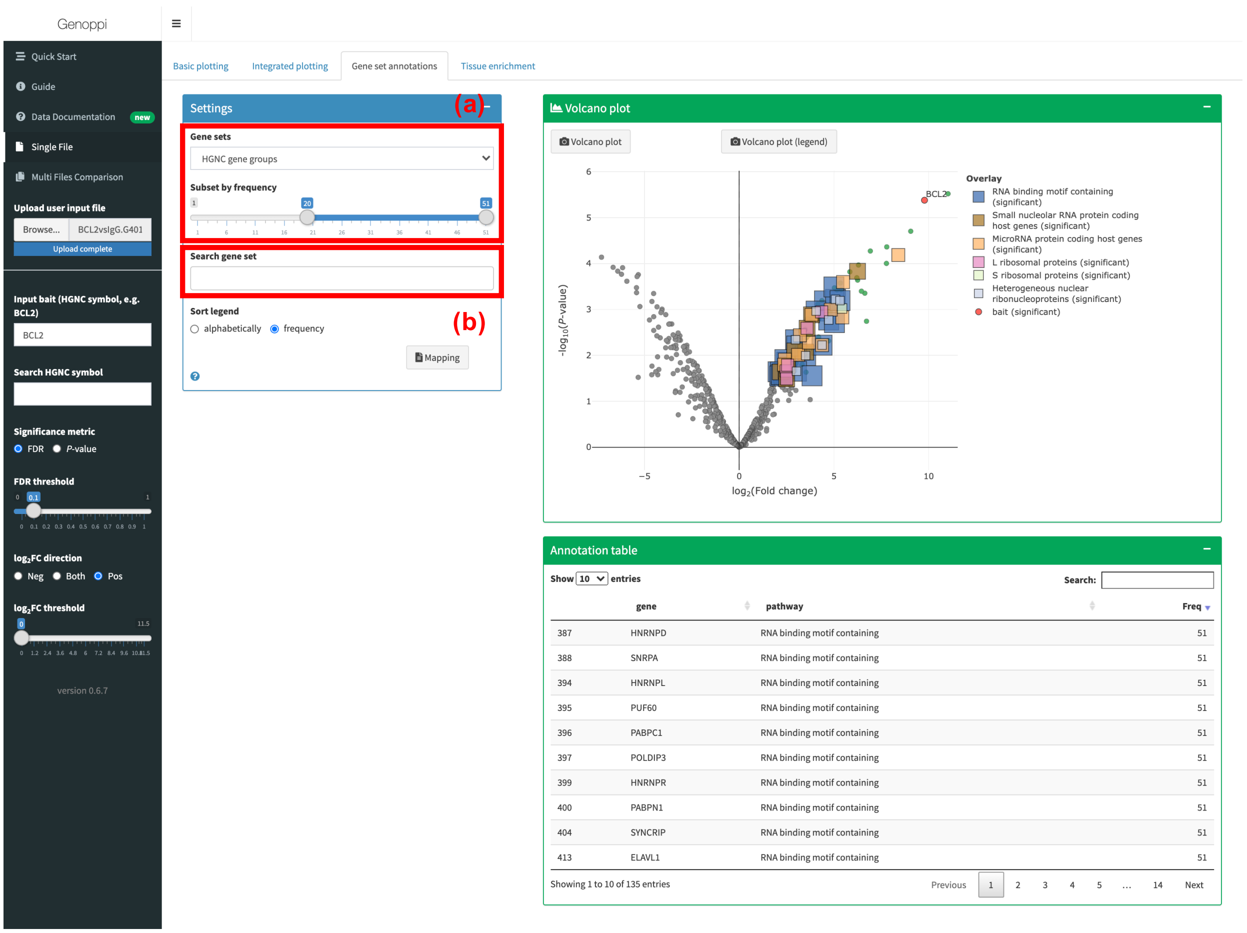

Screenshot 4. Gene set annotations interface showing significant proteins in proteomic data annotated with most recurrent HGNC gene groups.

Tissue enrichment

The “Tissue enrichment” module is an extension of the “GTEx or HPA” functionality described in the “Integrated plotting” module. Here, instead of integrating the proteomic data with a single tissue-specific gene list, Genoppi calculates the overlap enrichment of significant proteins in the proteomic data and tissue-specific genes across all tissues found in the “GTEx - RNA”, “GTEx - protein” or “HPA - RNA” dataset, which can be selected from a drop-down menu (**Screenshot 5a**). The hypergeometric enrichment results are displayed in a bar plot; the user can choose to display the -$\log_{10}$ *P*-value or -$\log_{10}$ *Q*-value (i.e. Benjamini-Hochberg FDR) on the x-axis, as well as selecting the significance threshold to highlight in the plot (**Screenshot 5b**). Hovering over each tissue’s bar in the plot would show the *P*-value, *Q*-value, and the list of significant proteins that are specifically expressed in the tissue.

In this module, the user also has the option of uploading sets of tissue- or cell-type-specific genes derived from their own expression data to perform the enrichment analysis (e.g. single-cell RNA-seq dataset that allows cell type deconvolution; **Screenshot 6a**). The data must be uploaded as a tab-delimited plain text file containing 3 columns: “tissue” (tissue or cell type name), “gene” (HGNC symbol), and “significant” (“TRUE” or “FALSE” indicating whether a gene is specifically expressed in the tissue or cell type). For example:

```
tissue      gene     significant
Celltype1   SHH      TRUE
Celltype1   UBC      TRUE
Celltype1   FOXP2    FALSE
Celltype2   RB1      TRUE
Celltype2   KRAS     FALSE
```

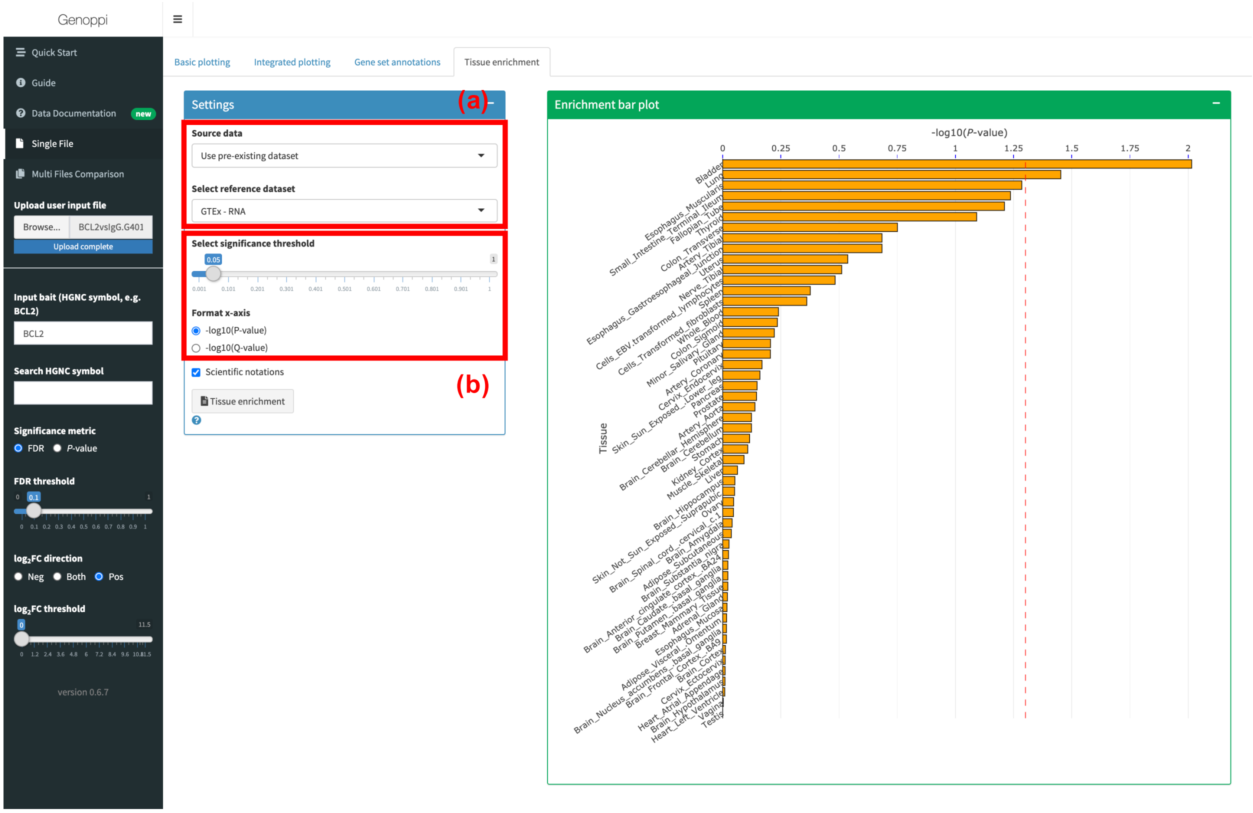

Screenshot 5. Tissue enrichment interface showing overlap enrichment of significant proteins in proteomic data and tissue-specific genes across GTEx or HPA tissues.

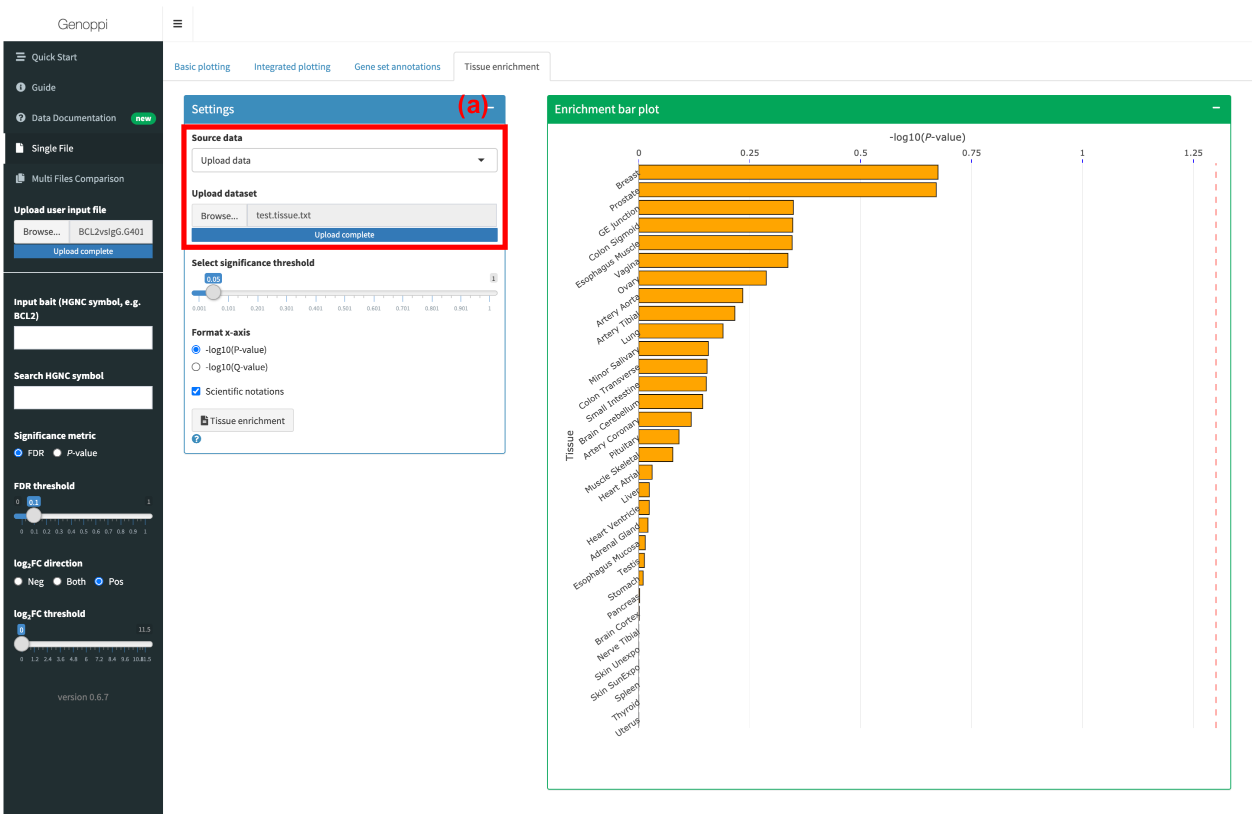

Screenshot 6. Tissue enrichment interface showing overlap enrichment of significant proteins in proteomic data and user-uploaded tissue- or cell-type-specific genes.

Multiple files comparison

Besides performing analyses for a single proteomic dataset as described in the previous sections, Genoppi also allows comparison of multiple proteomic datasets at once. Using the “Multi Files Comparison” input option, the user can upload two to three proteomic datasets to perform comparative analyses (**Screenshot 7a**). In the “Basic plotting” module, Genoppi would generate side-by-side volcano and scatter plots to compare the multiple datasets. The threshold for defining significant proteins can be individually adjusted for each dataset (**Screenshot 7b**). In the resulting plots, each significant protein is color-coded based on the combination of dataset files that share this significant protein. The possible combination groups are:

f1: significant proteins unique to file1 (red)
f2: significant proteins unique to file2 (yellow)
f3: significant proteins unique to file3 (blue)
f12: significant proteins identified in file1 and file2 (orange)
f13: significant proteins identified in file1 and file3 (purple)
f23: significant proteins identified in file2 and file3 (green)
f123: significant proteins identified in file1, file2, and file3 (white)

Furthermore, the “Venn diagrams” module summarizes the number of proteins in each combination group in a Venn diagram and displays the identities (i.e. HGNC symbols) of these proteins in a table (**Screenshot 8**).

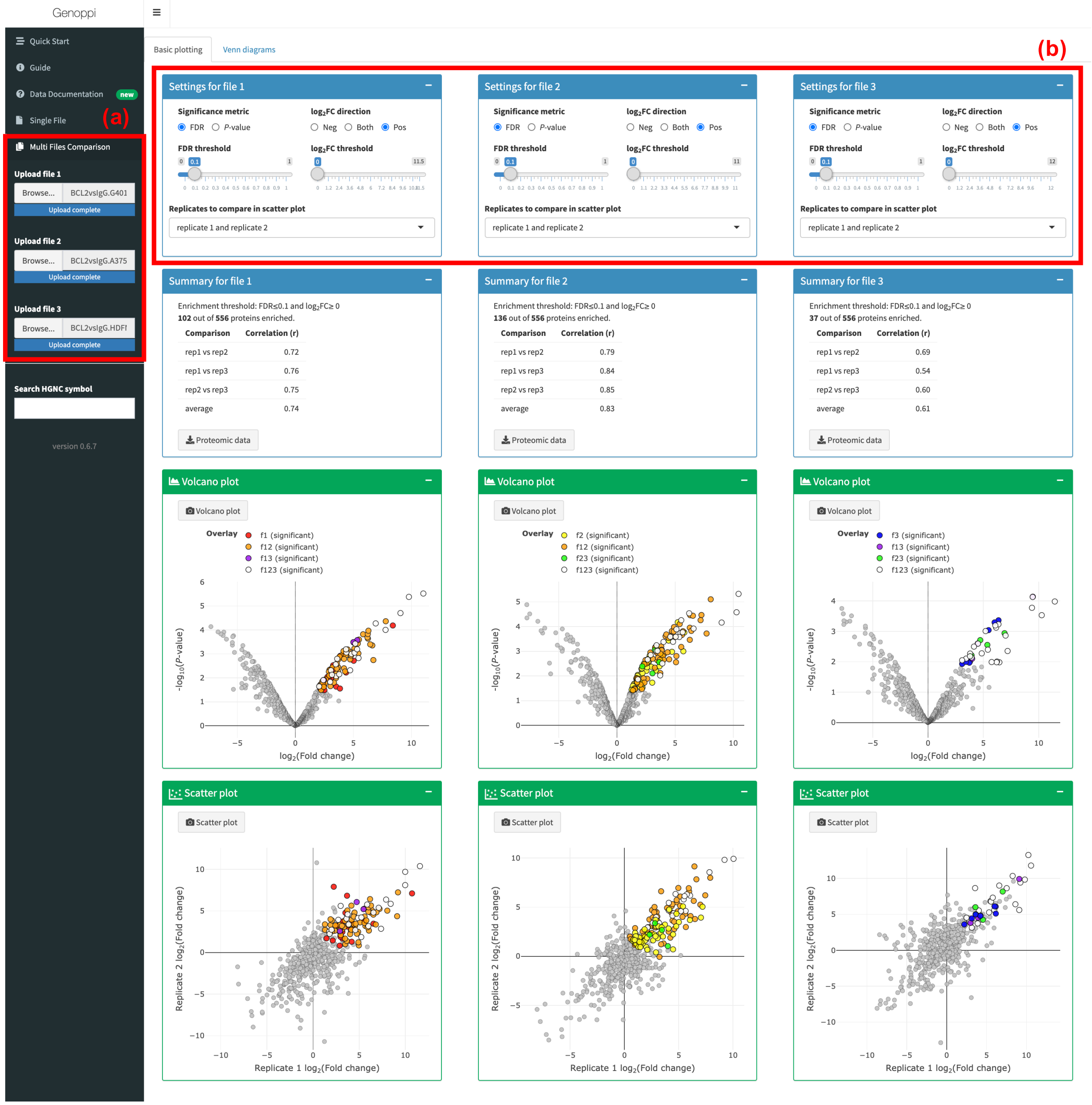

Screenshot 7. Basic plotting interface showing side-by-side volcano and replicate correlation scatter plots for multiple proteomic datasets.

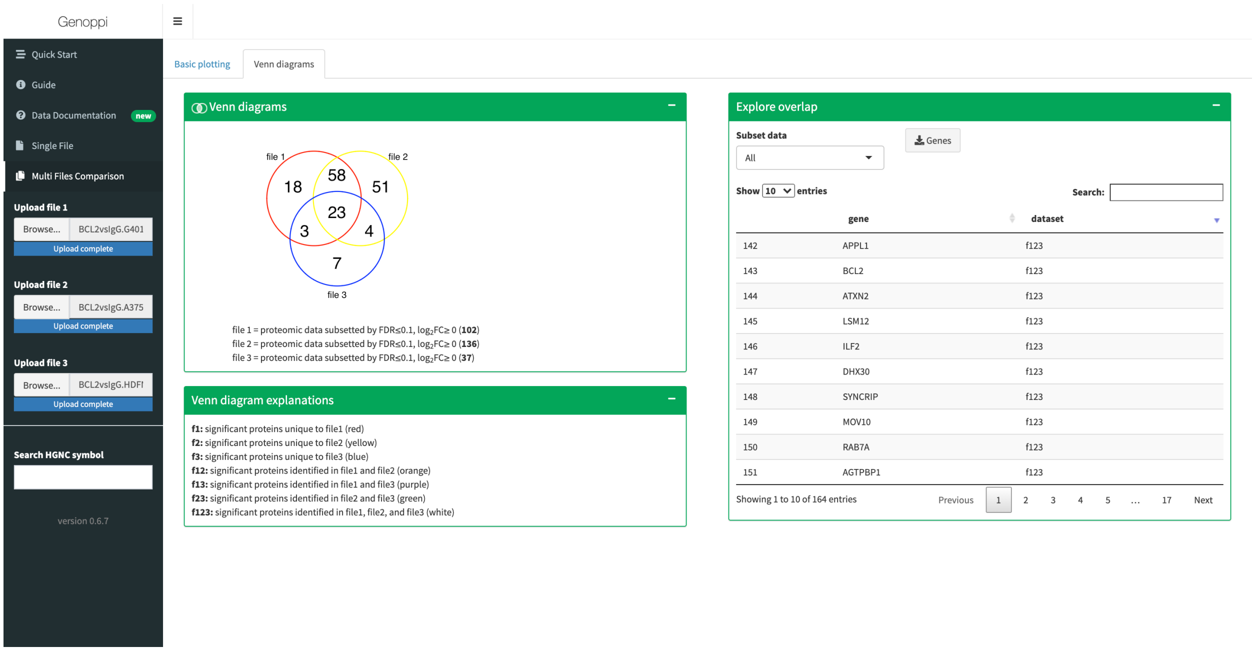

Screenshot 8. Venn diagrams interface showing overlaps of significant proteins identified in multiple proteomic datasets.

Downloads

Individual plots and data files generated by Genoppi can be downloaded in their respective modules by clicking on the interactive download buttons. In general, plots are saved as PNG image files, while text files are saved in comma-separated CSV format. Download buttons are only active once the relevant data have been generated.

Supplementary Note 2. Validation experiments of cell-type-specific protein interactions of TDP-43 in human neurons.

We conducted validation experiments and executed both i) western blots of identified interaction partners [to support the mass spectrometry data] and ii) reciprocal IPs of interaction partners of TDP-43 [to test the presence of TDP-43 in IPs of its interaction partners]. All experiments were executed in human GPiNs.

Western blots of TDP-43 interaction partners: We acquired and quality controlled 23 reagents (**Supplementary Data 1**) and tested 23 interactions found by LC-MS/MS by western blot (**Supplementary Fig. 4** and **Supplementary Data 6**). We observed a validation rate of 21/23 (or 91%) among interactors that span a wide range of $\log_2$ FC values in our IP dataset, indicating that proteins with both high and modest $\log_2$ FC can be confirmed by western blot to capture robust and reproducible interactions. We further note that this validation rate is concordant with an FDR of 0.1, where ~10% false positives are expected. Importantly, we were able to validate both known TDP-43 interactors in InWeb_InBioMap (10/11, or 91%; including all five of the tested ALS-relevant proteins) and newly identified interactors (11/12, or 92%) with similar success. Among the newly identified interactors, 8/9 (or 89%) interactors found in multiple cell lines and 3/3 (or 100%) interactors found only in GPiNs were validated. We also validated 17/19 (or 89%) interactors that had ≥ 2 imputed values prior to $\log_2$ FC calculation (see **Methods**); this finding supports that our imputation procedure generates biologically valid results.

Reciprocal IPs of TDP-43 interaction partners: We have executed reciprocal IPs of interaction partners of TDP-43, and tested the presence of TDP-43 in these IPs. We successfully immunoprecipitated five of the TDP-43 interactors validated by western blot in **Supplementary Fig. 4,** including three ALS-relevant proteins (MATR3, ATXN2, FUS) and two non-ALS proteins (RBMX and PARP1; **Supplementary Fig. 5a**; the IP of TDP-43 is included as a positive control). In the reciprocal IPs of ATXN2, FUS and RBMX, we see a clear band representing TDP-43 by western blot; whereas TDP-43 is faintly detected in the PARP1 IP and not at all in the MATR3 IP (**Supplementary Fig. 5b**). Together, this yields a validation rate of 3/5 (or 60%) to 4/5 (or 80%). We note that not all biologically valid interactions will lead to a positive result in a reciprocal IP experiment due to factors such as protein abundance and stoichiometric differences in a given cell type. For example, consider a protein X that has a highly specialized function in a cell and is present in low quantities, which interacts with protein Y that has a general function and is present

in high quantities. In a reciprocal IP of Y the presence of X may simply be ‘drowned out' by the many other interaction partners of Y (representing its wide array of functions), even when the IP of X clearly shows Y.

Overall, the validation rate in our IP westerns is 91% and the validation rate in our reciprocal IPs is 60-80%.

Supplementary Note 3. Discussion of MDM2 and PTEN IP-MS/MS results.

To exemplify the analytic features of Genoppi, we performed IPs of four proteins of interest (BCL2, TDP-43, MDM2, PTEN; hereafter called baits) in four distinct cell lines (GPiN, G401, T47D, A375; **Fig. 2a**) to generate proteomic data using label-free LC-MS/MS (**Methods**). The presence of the baits in IPs was confirmed by western blot analysis (**Supplementary Fig. 2a,** showing one biological replicate per bait). As expected, no band was detected for MDM2 in GPiNs, due to previously-characterized low neuronal expression of the *MDM2* gene. Although all four baits have been extensively investigated[19, 20, 21, 22, 23, 24], their characterization is often limited to a single cell type or disease model. The cell-type-specific protein-protein interactions observed for BCL2 and TDP-43 are described in the main text, while here we focus on the cell-type-specific interactomes of MDM2 and PTEN. Both proteins are known for their implication in cancer but have not been characterized in neurons[21, 22, 25, 26, 27].

MDM2 is an important negative regulator of the p53 tumor suppressor, both by inhibiting p53 transcriptional activation and targeting p53 for degradation by the proteasome[21]. More recently, it has been hypothesized that MDM2 is also able to function as an oncogene in p53-independent tumors. In order to investigate this possibility, we immunoprecipitated MDM2 in human epithelial cancer cell lines with a mutated p53 (T47D) and wild-type p53 (A375). The corresponding interactomes were resolved in LC-MS/MS and Genoppi was used to determine the correlation coefficients between IP replicates and the significance of MDM2 protein interactors when compared across the two cell lines (**Supplementary Fig. 2b** and **Supplementary Data 3**). We observed more proteins with significant $\log_2$ FC in A375 ($\log_2$ FC < 0 and FDR ≤ 0.1) than in T47D ($\log_2$ FC > 0 and FDR ≤ 0.1), which is in accordance with the close dependence between p53 and MDM2 in the cell. Interestingly, known MDM2 interactors catalogued in InWeb_InBioMap are evenly distributed across both cell lines and account for 41-43% of the significant proteins in each cell line (38/89 for A375, P = 0.85 using a hypergeometric distribution; 9/22 for T47D, P = 0.65; **Supplementary Fig. 2d**), suggesting that the absence of p53 does not detectably affect the known MDM2 interactome in the studied cancer cell lines. We further employed Genoppi to test for enrichment of cancer genes and found them to be predominantly present among interactors that are more specific of A375 cells with wild-type p53 (i.e., proteins with negative $\log_2$ FC), although they are not enriched among the significant proteins in A375 (P = 0.95; **Supplementary Fig. 2e**). This finding indicates that the newly identified interaction partners of MDM2 may depend on p53, a somewhat expected result considering the vast p53-dependent network of oncogenes

and the MDM2 regulatory role of p53. Future research should aim to interrogate whether the novel MDM2 interactions can be targeted to interfere with the MDM2-p53 relationship, which may have important implications for cancer therapy. Conversely, the thorough characterization of T47D-specific interactors is key to deciphering the p53-independent protein interaction network of MDM2.

PTEN (a well-known tumor suppressor gene that antagonizes phosphatidylinositol 3-phosphate kinase [PI3K] and AKT signaling) encodes for a phosphatase and is mutated in a large number of cancers at a high frequency[22]. Interestingly, germline mutations of PTEN have also been described in a subset of patients with autism spectrum disorders and macrocephaly[26]. However, it is unclear whether the role of PTEN in cell proliferation is also implicated in these neurodevelopmental phenotypes[26, 27]. To investigate this possibility, we used Genoppi to compare the interactomes of PTEN in human cancer cells (G401) and neurons (GPiNs; **Supplementary Fig. 2c** and **Supplementary Data 3**). Only a small portion of the significant proteins in each cell line ($\log_2$ FC < 0 and FDR ≤ 0.1 for G401; $\log_2$ FC > 0 and FDR ≤ 0.1 for GPiN) are known PTEN interactors in InWeb_InBioMap (**Supplementary Fig. 2f**). In particular, only seven out of the 48 significant proteins for GPiN are known interactors, suggesting that PTEN has limited interactions with its classic partners in human neurons. Next, we used Genoppi to test the representation of cancer genes among significant proteins in both cell lines. In line with the expectation that PTEN interaction partners are linked to proliferation in both cell types, we did not observe a significant difference between G401 (7/212, P = 0.53) and GPiN (1/48, P = 0.80; **Supplementary Fig. 2g**). Genoppi was also used to assess the enrichment of proteins encoded by neuropsychiatric disease genes linked to autism and schizophrenia (**Supplementary Fig. 2h**). We found a slightly higher proportion of risk gene products among significant proteins in GPiNs (8.3%, or 4/48) compared to in G401 cells (2.4%, or 5/212); follow-up studies are necessary to elucidate whether the risk proteins interacting with PTEN in GPiNs are part of the pathways driving the neuron-specific function of PTEN. This result highlights the importance of studying and comparing both cell-type-specific and shared interactions of PTEN in cancer cells and human neurons to yield insights into its specific function in neurodevelopment and disease.

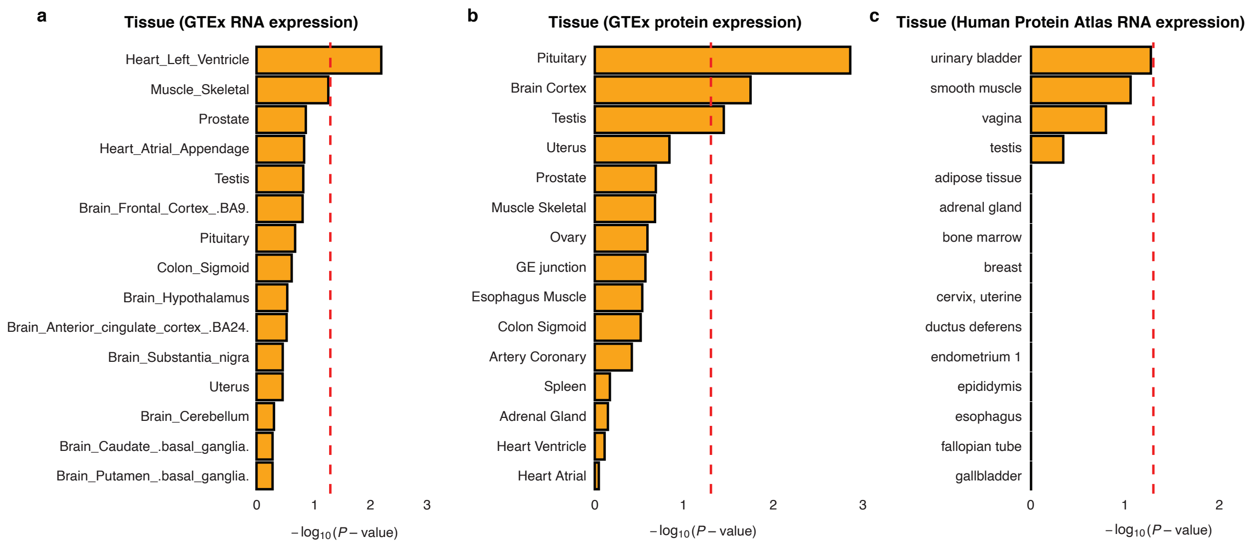

Supplementary Figure 1. Tissue enrichment of significant ($\log_2$ FC > 0 and FDR ≤ 0.1) interactors in published CRBN interaction data (in MM1S multiple myeloma cells versus control samples; related to **Figure 1**). One-tailed hypergeometric tests were used to assess the significance of overlap between CRBN interactors and tissue-specific genes derived from GTEx RNA expression **(a)**, GTEx protein expression **(b)**, or HPA RNA expression **(c)** data. Nominal *P*-values are plotted, with vertical dashed red line indicating the nominal significance ($P < 0.05$) threshold; exact *P*-values are provided in the Source Data file.

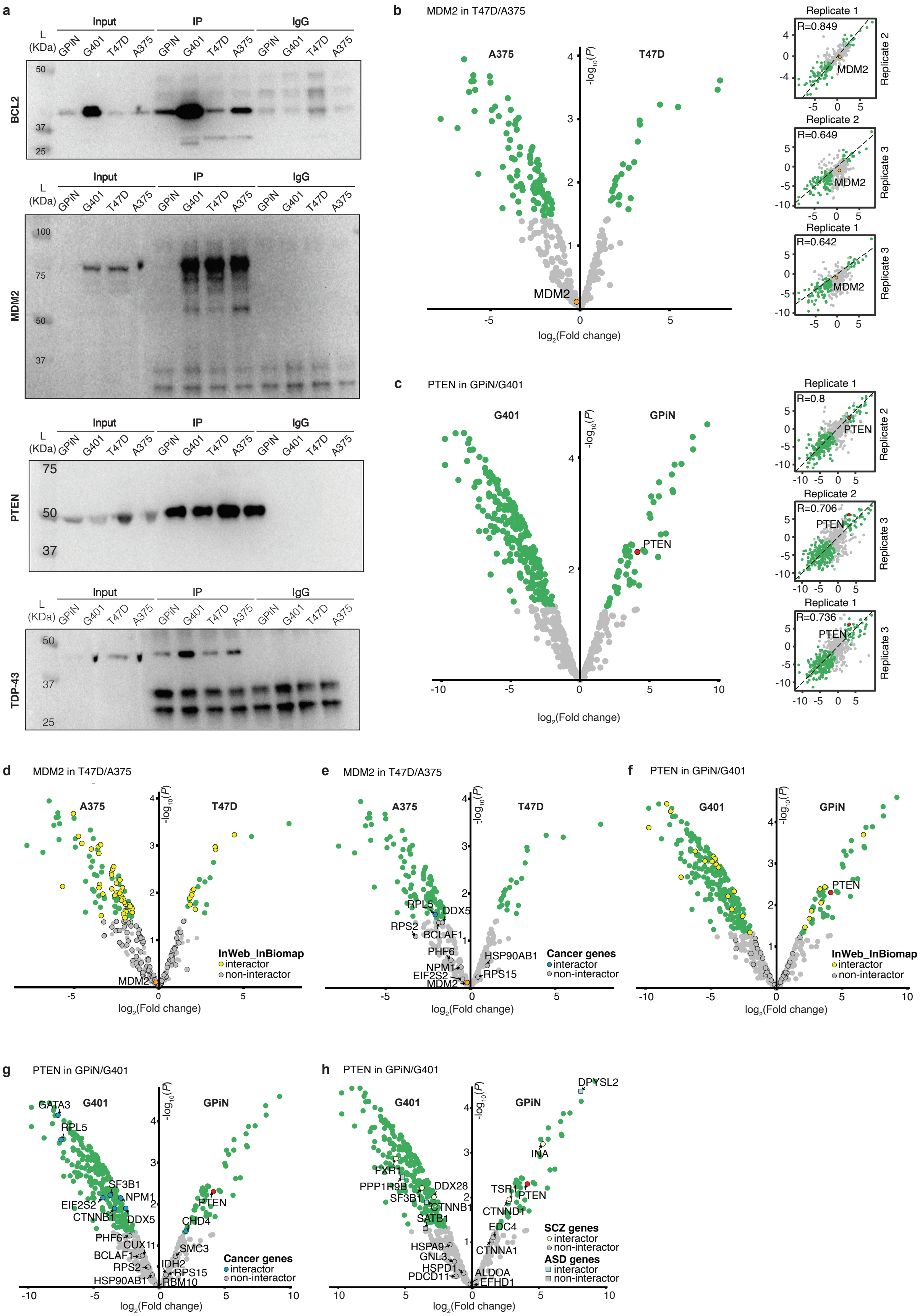
a
Input
IP
IgG
L (KDa)
GPiN
G401
T47D
A375
BCL2
MDM2
PTEN
TDP-43
50
37
25
100
75
b
MDM2 in T47D/A375
A375
T47D
MDM2
-log10(P)
log2(Fold change)
Replicate 1
Replicate 2
Replicate 3
R=0.849
R=0.649
R=0.642
c
PTEN in GPiN/G401
G401
GPiN
PTEN
R=0.8
R=0.706
R=0.736
d
MDM2 in T47D/A375
InWeb_InBiomap
interactor
non-interactor
e
MDM2 in T47D/A375
RPL5
DDX5
RPS2
BCLAF1
PHF6
NPM1
EIF2S2
HSP90AB1
RPS15
Cancer genes
interactor
non-interactor
f
PTEN in GPiN/G401
g
PTEN in GPiN/G401
GATA3
RPL5
SF3B1
NPM1
EIF2S2
CTNNB1
DDX5
PHF6
CUX1
BCLAF1
RPS2
HSP90AB1
CHD4
SMC3
IDH2
RPS15
RBM10
h
PTEN in GPiN/G401
DPYSL2
INA
FXR1
PPP1R9B
DDX28
SF3B1
CTNNB1
SATB1
TSR1
CTNND1
EDC4
CTNNA1
HSPA9
GNL3
HSPD1
PDCD11
ALDOA
EFHD1
SCZ genes
ASD genes

Supplementary Figure 2. Additional IP-MS/MS analysis results (related to **Figure 2**). **(a)** BCL2, MDM2, PTEN and TDP-43 were immunoprecipitated in four cell lines (GPiN, G401, T47D, A375) and detected by Western blot. Each blot is representative of three IP replicates. IN, input; IP, immunoprecipitation; IgG, IgG isotype control. Source data are provided as a Source Data file. **(b)** MDM2 IP results in T47D versus A375 cells. Left: volcano plot showing average $\log_2$ fold change (FC) and corresponding $-\log_{10}$ *P*-value of each identified protein. Right: scatter plots showing reproducibility of three IP replicates in terms of $\log_2$ FC correlation; Pearson's correlation (r) is reported in each plot. The bait (MDM2), significant interactors (FDR ≤ 0.1), and other identified proteins are shown in orange, green, and grey, respectively. **(c)** PTEN IP results in GPiN versus G401 cells. Same layout as (b), except the bait (PTEN) is shown in red. **(d)** Volcano plot from (b) overlaid with known MDM2 interactors in InWeb_InBioMap (black border circles). Overlap enrichment *P* = 0.85 and 0.65 for significant proteins in A375 ($\log_2$ FC < 0 and FDR ≤ 0.1) and T47D ($\log_2$ FC > 0 and FDR ≤ 0.1), respectively. **(e)** Volcano plot from (b) overlaid with proteins encoded by cancer genes (black border circles). Overlap enrichment *P* = 0.95 and 1 for significant proteins in A375 and T47D, respectively. **(f)** Volcano plot from (c) overlaid with known PTEN interactors in InWeb_InBioMap (black border circles). Overlap enrichment *P* = 0.99 and 0.22 for significant proteins in G401 and GPiN, respectively. **(g)** Volcano plot from (c) overlaid with proteins encoded by cancer genes (black border circles). Overlap enrichment *P* = 0.53 and 0.80 for significant proteins in G401 and GPiN, respectively. For (d)-(g), overlap enrichment *P*-values were calculated using one-tailed hypergeometric tests. **(h)** Volcano plot from (c) overlaid with proteins encoded by neuropsychiatric disease genes (black border circles or squares for schizophrenia or autism genes, respectively). Overlap enrichment was not calculated as part of this gene list was mapped from GWAS SNPs using linkage disequilibrium information.

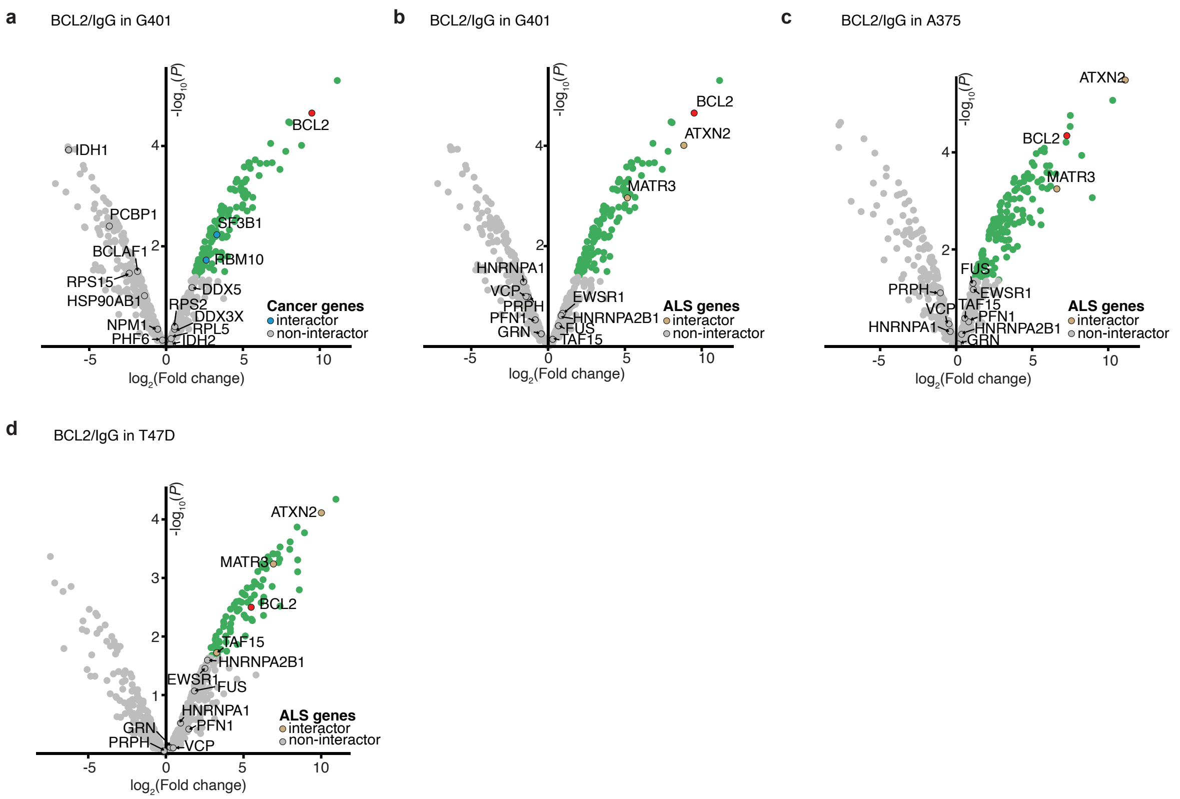

Supplementary Figure 3. Volcano plots of BCL2 versus IgG control IP results in cancer cells, overlaid with proteins encoded by cancer or ALS genes (related to **Figure 2**). **(a)** IP results in G401 cells overlaid with cancer genes (overlap enrichment P = 0.84). **(b)** IP results in G401 cells overlaid with ALS genes (overlap enrichment P = 0.72). **(c)** IP results in A375 cells overlaid with ALS genes (overlap enrichment P = 0.84). **(d)** IP results in T47D cells overlaid with ALS genes (overlap enrichment P = 0.28). The bait (BCL2), interactors ($\log_2$ FC > 0 and FDR ≤ 0.1), and non-interactors are shown in red, green, and grey, respectively. Proteins encoded by cancer genes (a) or ALS genes (b-d) are marked by black border circles. Overlap enrichment P-values were calculated using one-tailed hypergeometric tests.

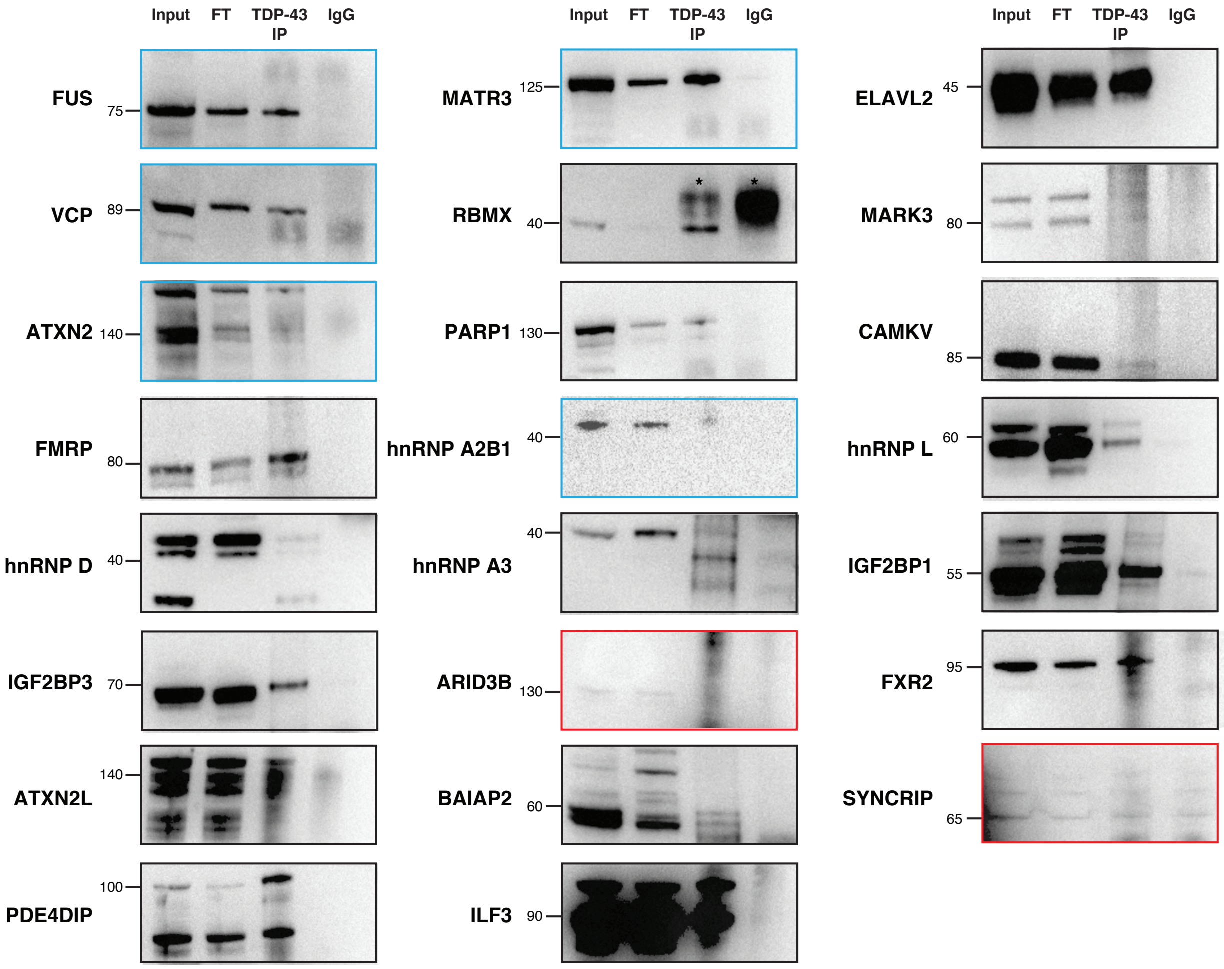

Supplementary Figure 4. Western blots of TDP-43 immunoprecipitations in human GPiNs. Western blots were performed using antibodies against the proteins named to the left of each blot after immunoprecipitation of TDP-43 in GPiNs. Western blots that successfully validate significant interactions identified by LC-MS/MS are framed in black (non-ALS risk genes) and blue (ALS-risk genes), and negative blots (ARID3B, SYNCRIP) are framed in red. Each blot represents a TDP-43 immunoprecipitation that is independent of the three IP replicates subjected to MS. Asterisks (*) indicate the IgG heavy-chain and molecular weights (KDa) that are closest to the ones of the detected proteins are also indicated. IN, input; FT, flow-through; IP, immunoprecipitation; IgG, IgG isotype control. Source data are provided as a Source Data file.

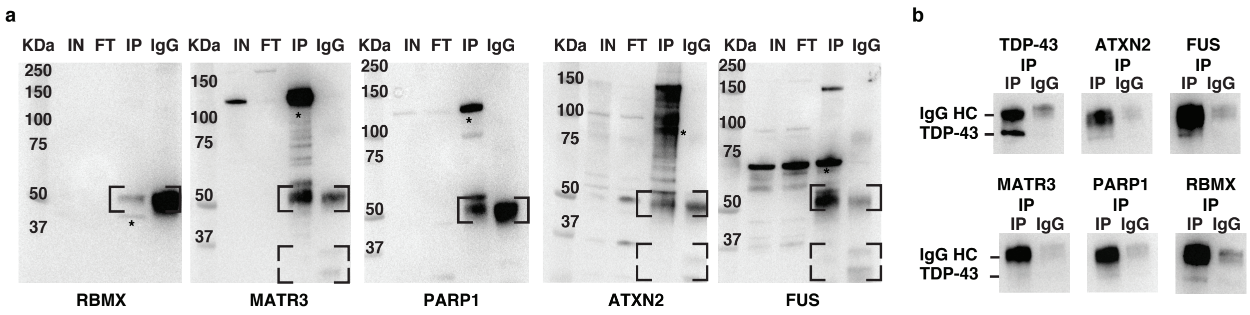

Supplementary Figure 5. (a) Western blots showing successful immunoprecipitations of RBMX, MATR3, PARP1, ATXN2 and FUS in human GPiNs. Asterisks (*) indicate the main immunoprecipitated isoform and open boxes indicate the IgG heavy and light chains (if detected). Each blot represents one of two IP replicates. KDa, kiloDaltons; IN, input; FT, flow-through; IP, immunoprecipitation; IgG, IgG isotype control. **(b)** Western blots showing co-immunoprecipitation of TDP-43 in IPs from (a). Expected bands corresponding to TDP-43 and the IgG heavy-chain (HC) are indicated in the figure. Clear TDP-43 enrichment can be observed in its own IP, as well as in ATXN2, FUS and RBMX IPs. A faint band can be observed in the PARP1 IP. The TDP-43 IP blot represents a TDP-43 immunoprecipitation that is independent of the three IP replicates subjected to MS; other IP blots represent one of two IP replicates shown in (a). IP, immunoprecipitation; IgG, IgG isotype control. Source data are provided as a Source Data file.

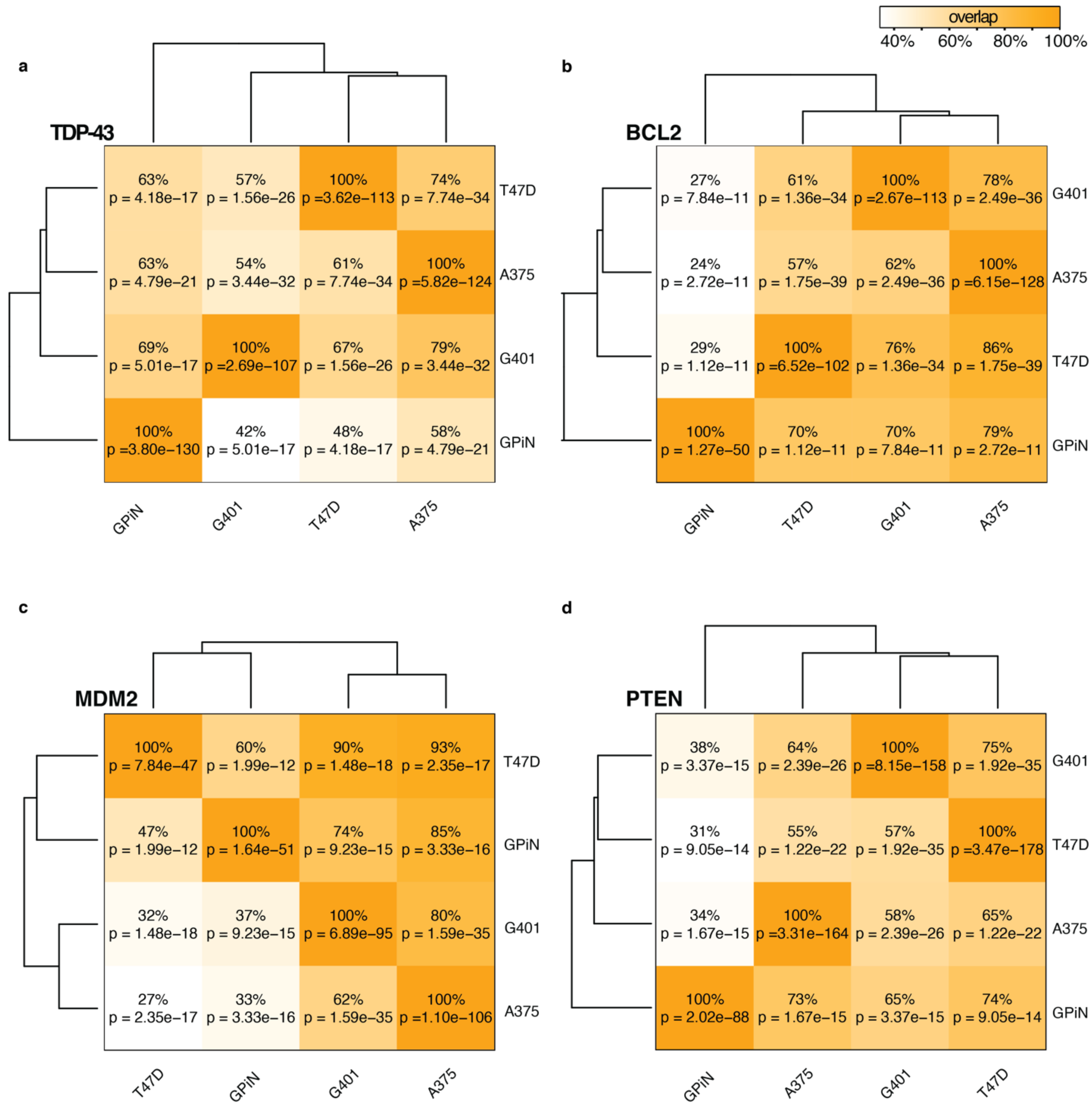

Supplementary Figure 6. Clustered heat maps indicating the percentage of overlap between significant interactors ($\log_2$ FC > 0 and FDR ≤ 0.1 when compared against IgG control) of TDP-43 **(a)**, BCL2 **(b)**, MDM2 **(c)**, and PTEN **(d)** across different cell lines. The overlap percentages were calculated using the row cell line as reference (i.e., the denominator); the corresponding nominal *P*-values were calculated using a one-tailed hypergeometric test. Dendrograms were generated from hierarchical clustering of the overlap percentages.

Supplementary References

1. Yates B, Braschi B, Gray KA, Seal RL, Tweedie S, Bruford EA. Genenames.org: the HGNC and VGNC resources in 2017. *Nucleic Acids Res* **45**, D619-D625 (2017).
2. The UniProt Consortium. UniProt: the universal protein knowledgebase. *Nucleic Acids Res* **46**, 2699 (2018).
3. Ritchie ME*, et al.* limma powers differential expression analyses for RNA-sequencing and microarray studies. *Nucleic Acids Res* **43**, e47 (2015).
4. Lage K*, et al.* A human phenome-interactome network of protein complexes implicated in genetic disorders. *Nat Biotechnol* **25**, 309-316 (2007).
5. Li T*, et al.* A scored human protein-protein interaction network to catalyze genomic interpretation. *Nat Methods* **14**, 61-64 (2017).
6. Razick S, Magklaras G, Donaldson IM. iRefIndex: a consolidated protein interaction database with provenance. *BMC Bioinformatics* **9**, 405 (2008).
7. Huttlin EL*, et al.* The BioPlex Network: A Systematic Exploration of the Human Interactome. *Cell* **162**, 425-440 (2015).
8. Huttlin EL*, et al.* Dual Proteome-scale Networks Reveal Cell-specific Remodeling of the Human Interactome. Preprint at https://wwwbiorxivorg/content/101101/20200119905109v1 (2020).
9. Buniello A*, et al.* The NHGRI-EBI GWAS Catalog of published genome-wide association studies, targeted arrays and summary statistics 2019. *Nucleic Acids Res* **47**, D1005-D1012 (2019).
10. Karczewski KJ*, et al.* The mutational constraint spectrum quantified from variation in 141,456 humans. *Nature* **581**, 434-443 (2020).
11. Finucane HK*, et al.* Heritability enrichment of specifically expressed genes identifies disease-relevant tissues and cell types. *Nat Genet* **50**, 621-629 (2018).
12. Jiang L*, et al.* A Quantitative Proteome Map of the Human Body. *Cell* **183**, 269-283 e219 (2020).
13. Uhlen M*, et al.* Proteomics. Tissue-based map of the human proteome. *Science* **347**, 1260419 (2015).
14. The 1000 Genomes Project Consortium*.* A global reference for human genetic variation. *Nature* **526**, 68-74 (2015).
15. Ashburner M*, et al.* Gene ontology: tool for the unification of biology. The Gene Ontology Consortium. *Nat Genet* **25**, 25-29 (2000).

16. The Gene Ontology Consortium. The Gene Ontology Resource: 20 years and still GOing strong. *Nucleic Acids Res* **47**, D330-D338 (2019).
17. Subramanian A*, et al.* Gene set enrichment analysis: a knowledge-based approach for interpreting genome-wide expression profiles. *Proc Natl Acad Sci U S A* **102**, 15545-15550 (2005).
18. Liberzon A, Birger C, Thorvaldsdottir H, Ghandi M, Mesirov JP, Tamayo P. The Molecular Signatures Database (MSigDB) hallmark gene set collection. *Cell Syst* **1**, 417-425 (2015).
19. Umoh ME*, et al.* A proteomic network approach across the ALS-FTD disease spectrum resolves clinical phenotypes and genetic vulnerability in human brain. *EMBO Mol Med* **10**, 48-62 (2018).
20. Freibaum BD, Chitta RK, High AA, Taylor JP. Global analysis of TDP-43 interacting proteins reveals strong association with RNA splicing and translation machinery. *J Proteome Res* **9**, 1104-1120 (2010).
21. Moll UM, Petrenko O. The MDM2-p53 interaction. *Mol Cancer Res* **1**, 1001-1008 (2003).
22. Lee YR, Chen M, Pandolfi PP. The functions and regulation of the PTEN tumour suppressor: new modes and prospects. *Nat Rev Mol Cell Biol* **19**, 547-562 (2018).
23. Yip KW, Reed JC. Bcl-2 family proteins and cancer. *Oncogene* **27**, 6398-6406 (2008).
24. Kaya-Aksoy E*, et al.* The pro-apoptotic Bcl-2 family member Harakiri (HRK) induces cell death in glioblastoma multiforme. *Cell Death Discov* **5**, 64 (2019).
25. Nag S, Qin J, Srivenugopal KS, Wang M, Zhang R. The MDM2-p53 pathway revisited. *J Biomed Res* **27**, 254-271 (2013).
26. Varga EA, Pastore M, Prior T, Herman GE, McBride KL. The prevalence of PTEN mutations in a clinical pediatric cohort with autism spectrum disorders, developmental delay, and macrocephaly. *Genet Med* **11**, 111-117 (2009).
27. Tilot AK, Frazier TW, 2nd, Eng C. Balancing Proliferation and Connectivity in PTEN-associated Autism Spectrum Disorder. *Neurotherapeutics* **12**, 609-619 (2015).
