# Supplementary material for: Genoppi is an open-source software for robust and standardized integration of proteomic and genetic data
Source: Nat Commun. 2021 May 10;12:2580. doi: 10.1038/s41467-021-22648-5 (PMC8110583; doi:10.1038/s41467-021-22648-5)
Supplement: Supplementary file 2 — Description of Additional Supplementary Files [file 41467_2021_22648_MOESM2_ESM.docx]

**Description of Additional Supplementary Files**

File Name: Supplementary Data 1

Description: Antibodies used in this study.

File Name: Supplementary Data 2

Description: Processed IP-MS data generated in this study.

File Name: Supplementary Data 3

Description: Summary of Genoppi analysis results for IP-MS data of 4 baits across 4 cell lines.

File Name: Supplementary Data 4

Description: Gene lists used for integrative analyses in Genoppi.

File Name: Supplementary Data 5

Description: Pathway overrepresentation analysis of cell-type-specific TDP-43 interactors using MSigDB Reactome pathways.

File Name: Supplementary Data 6

Description: Summary of TDP-43 interactors in GPiNs tested in validation experiments.

File Name: Supplementary Data 7

Description: Comparison of Genoppi vs. SAINTexpress analysis results for IP-MS data of 4 baits across 4 cell lines.
